# SIRT1 mediated autophagy enhancement by *Lactobacillus fermentum* derived oligosaccharides accelerates wound healing in biofilm associated infection

**DOI:** 10.1038/s41598-025-30280-2

**Published:** 2025-12-26

**Authors:** Amany E. Ragab, Lamiaa A. Al-Madboly, Ghada M. Al-Ashmawy, Mariam A. Abo-Saif

**Affiliations:** 1 Department of Pharmacognosy, Faculty of Pharmacy, Tanta University, Tanta, 31527 Egypt; 2 Department of Microbiology and Immunology, Faculty of Pharmacy, Tanta University, Tanta, 31527 Egypt; 3 Department of Biochemistry, Faculty of Pharmacy, Tanta University, Tanta, 31527 Egypt; 4 Department of Biochemistry, Faculty of Pharmacy, Alsalam University, Kafr Alzayat, 31611 Algharbia Egypt

**Keywords:** Oligosaccharide, Bacterial exopolysaccharides, NMR spectroscopy, Multidrug-resistant *Pseudomonas aeruginosa*, Wound healing, Sirtuin 1 (SIRT1), Autophagy, *Beclin 1*, *ATG5*, Biochemistry, Microbiology, Medical research

## Abstract

The present study explores the structural elucidation, antimicrobial properties, and wound-healing potential of a novel oligosaccharide, OligoF, derived from *Lactobacillus fermentum*. Structural analysis using paper chromatography, and 1D & 2D NMR spectroscopy indicated that OligoF is a linear tetrasaccharide comprising β-D-glucose, α-L-rhamnose, and D-glucuronic acid in a defined sequence. This structural elucidation was further confirmed by key chemical shifts, coupling constants, and glycosidic linkage assignments. OligoF exhibited potent antibacterial activity against multidrug-resistant *Pseudomonas aeruginosa* isolates, demonstrating inhibition zones of 60–63 mm, a minimum inhibitory concentration (MIC) of 16 µg/mL, and a minimum bactericidal concentration (MBC) of 32 µg/mL. Biofilm eradication assays revealed a significant reduction in biofilm viability by ~ four-fold at sub-MIC concentrations, as evidenced by scanning electron microscopy. OligoF-treated biofilms displayed notable disruption of extracellular matrix and severe bacterial cell distortion compared to untreated controls. In vivo wound-healing assays in rats showed that OligoF significantly reduced wound area and enhanced skin repair compared to untreated controls. Treatment with OligoF notably increased the concentration and gene expression of SIRT1 as well as upregulated the gene expression of *beclin1* and *ATG5* which are critical regulators of autophagy and cellular repair processes. Histopathological analysis corroborated these findings, revealing enhanced re-epithelialization, granulation tissue formation, and vascularized connective tissue deposition in OligoF-treated wounds. These findings underscore the potential of OligoF as a multifunctional agent with antibacterial and wound-healing properties, paving the way for its application in managing multidrug-resistant infections and promoting tissue repair.

**Supplementary Information:**

The online version contains supplementary material available at 10.1038/s41598-025-30280-2.

## Introduction

*Pseudomonas aeruginosa* is a Gram-negative, motile, aerobic, opportunistic pathogen with intrinsic resistance to many antimicrobials. Consequently, infections caused by *P. aeruginosa* are often life-threatening, leading to prolonged hospital stays, increased morbidity, and elevated mortality rates^1^. A major virulence factor of this pathogen is its ability to form biofilms, wherein bacterial cells are embedded within a self-produced extracellular matrix. This biofilm lifestyle enhances colonization of diverse surfaces and confers tolerance to host immune responses and antimicrobial therapies^2^. Biofilm-associated infections, therefore, represent a major clinical challenge, particularly in wounds where they impair host defenses and delay tissue repair.

Wound healing is a fundamental physiological process that preserves skin integrity and function. Impaired wound repair, manifesting as pathological scarring or chronic non-healing wounds, is a significant global health burden^3^. In wounds colonized by biofilm-forming pathogens such as *P. aeruginosa*, the healing process is further compromised, underscoring the need to better understand host–pathogen interactions that sustain biofilm persistence and to identify novel therapeutic approaches^1^.

Autophagy is an evolutionarily conserved process that degrades and recycles intracellular components, enabling cells to adapt to stress and resist unfavorable conditions. Beyond its homeostatic functions, autophagy regulates inflammation, apoptosis, oxidative stress, and immune signaling, and it is implicated in the pathogenesis of numerous diseases^4^. Importantly, autophagy also serves as a host defense mechanism against intracellular and biofilm-forming pathogens, facilitating bacterial clearance while limiting excessive inflammation^5^. During wound healing, autophagy contributes to distinct phases of tissue repair: it supports infection clearance and modulates inflammation in the inflammatory phase, while in the proliferative phase it prevents apoptosis, reduces oxidative stress, and promotes cell survival and re-epithelialization^6,7^.

Sirtuin 1 (SIRT1), a NAD⁺-dependent class III histone deacetylase, is a key regulator of autophagy, metabolism, stress responses, and immune signaling^8,9^. In wound repair, SIRT1 influences inflammation, epidermal differentiation, and keratinocyte migration^10^. Upregulation of the gene encoding SIRT1 is associated with enhanced autophagy, promoted bacterial clearance, attenuated inflammatory responses, and supported tissue regeneration. Conversely, reduced expression or dysregulation of SIRT1–autophagy signaling may favor pathogen persistence and facilitate biofilm maturation, thereby impairing wound healing^9,10^.

Microbial oligosaccharides have attracted growing attention for their diverse biological activities, including antimicrobial, anti-inflammatory, and immunomodulatory effects. These compounds may influence host defense pathways as well as microbial colonization, making them promising candidates for the control of infections and wound healing. However, their impact on host signaling pathways during biofilm-associated infections remains poorly defined^11,12^. Accordingly, we hypothesized that OligoF, a novel microbial oligosaccharide under investigation, could promote wound healing by modulating this signaling axis in the presence of biofilm-forming pathogens. The present study, therefore, aimed to evaluate the effects of OligoF on wound healing, to compare its efficacy with povidone-iodine (10%) in infected wounds, and to investigate the underlying mechanisms with a particular focus on the interplay between microbial oligosaccharides, SIRT1–autophagy signaling, and biofilm colonization.

## Materials and methods

### Bacterial strains and culture conditions

#### *Lactobacillus fermentum* as a source for the oligosaccharides

This strain was obtained from the Department of Microbiology and Immunology, Faculty of Pharmacy, Tanta University, Egypt. For inoculation, 1 mL of *L. fermentum* overnight culture, containing approximately 4.0 × 10^8^ CFU/mL, was transferred into De Man-Rogosa-Sharpe (MRS) broth. The culture was incubated at 37 °C under anaerobic conditions for 24 to 48 h. The bacterial cells were harvested by centrifugation at 10,000 rpm for 30 min at 2 °C. The culture supernatant was carefully separated and filtered using a 0.22 µm pore-size membrane filter^11,12^.

### Test pathogens and culture conditions

Ten clinical isolates of MDR *P. aeruginosa* were used in the present study. They were obtained from the Department of Microbiology and Immunology, Faculty of Pharmacy, Tanta University, Egypt. Test isolates were formerly identified by Abdelaziz et al.^13^.

### Reference strain

*Pseudomonas aeruginosa* ATCC 27,853, a quality control strain, was used in this study. It was obtained from the Department of Microbiology and Immunology, Faculty of Pharmacy, Tanta University, Tanta, Egypt.

### Extraction and purification of the oligosaccharide (OligoF)

*Lactobacillus fermentum* (~ 4.0 × 10^8^ CFU/mL) was cultured in MRS broth and incubated at 37 °C under anaerobic conditions for 24–48 h. Bacterial cells were harvested by centrifugation at 10,000 rpm for 30 min at 2 °C, and the culture supernatant was separated and filtered through a 0.22 µm membrane. The extracellular mixture produced by the bacterial culture was first treated with 10% trichloroacetic acid (1:1, v/v) to precipitate proteins, and the resulting suspension was centrifuged at 10,000 rpm for 30 min at 2 °C to remove the pellet. The clarified supernatant was then subjected to three successive rounds of cold ethanol precipitation (3:1, v/v). The oligosaccharides were collected by centrifugation at 10,000 rpm for 30 min at 2 °C, and the ethanol-soluble fraction was concentrated under reduced pressure using a rotary evaporator at 40 °C. The purified oligosaccharide fraction obtained through this process was designated as OligoF and stored at 4 °C until further analysis^11,12^.

### NMR and mass analysis of OligoF

1D and 2D proton and carbon NMR were run using a spectrophotometer Bruker Avance 400 (Karlsruh, Germany) at 400 MHz and D_2_O or DMSO as a solvent.

### Complete hydrolysis of OligoF and study of the monomeric composition

The hydrolysis process followed a published procedure^14^. OligoF (200 mg) was dissolved in 1N H_2_SO_4_ (10 mL) and heated in a boiling water bath for 24 h using an air condenser. At the end of the hydrolysis time, the solution was cooled down to room temperature and neutralized using solid barium carbonate, then filtered using filter paper. The precipitate was washed twice with distilled water (5 mL) and the combined filtrate was concentrated to 2 mL in an oven at 40 °C. To the concentrated filtrate, 3 volumes of absolute alcohol were added to precipitate uronic acids which were separated by centrifugation at 4000 rpm leaving non-uronic acid monomers in the solution. The uronic acid precipitated was reconstituted in 1 mL distilled water. The monomeric composition of OligoF was determined by running paper chromatography for the uronic and non-uronic acid monomers alongside authentic monosaccharides (β-D-glucose, β-D-galactose, α-L-rhamnose, α-L-arabinose, D-xylose, D-galacturonic acid, and D-glucuronic acid) using the solvent system *n*-butanol/acetic acid/water (4:1:5) and visualized as brown spots by aniline acid phthalate spray after heating at 110 °C. The authentic sugars were obtained from Sigma Aldrich except D-glucuronic acid which was prepared from an authentic D-glucuronic acid lactone by dissolving 5 mg in 1N NaOH and spontaneous hydrolysis of the ester linkage occurs to give D-glucuronic acid.

### Stepwise hydrolysis screening of OligoF

The same procedure as in complete hydrolysis was employed on a larger scale. OligoF (2 g) dissolved in 100 mL of 1N H_2_SO_4_ was heated in a boiling water bath using air condenser and samples (10 mL, each) were withdrawn on intervals (15 min, 30 min, 45 min, 60 min, 75 min, 90 min and 120 min). The samples were treated as previously detailed in the complete hydrolysis step^14^.

### Screening for antibacterial activity

The agar well-diffusion test was used for preliminary assessment of the antibacterial activity of OligoF against all MDR *P. aeruginosa* isolates as previously described by Al-Madboly and Abdullah^15^. Briefly, about 10 mg/mL of the test OligoF was dissolved in sterile distilled water, followed by filter-sterilization through a 0.22-μm-pore-size filter. Next, an overnight culture of each strain was diluted to give 1 × 10^6^ cells/mL, then 100 μL was transferred to Muller-Hinton Agar (MHA) to form seeded media. After that, a sterile corkborer (6-mm diameter) was utilized to make wells in agar plates. Next, OligoF solution was used to fill the wells. All plates were incubated for 16–18 h at 35 ± 2 °C, and then the diameters of growth inhibition zones were determined in mm. Each measurement indicated the average of three independent experiments, and standard deviations were calculated.

### Broth microdilution susceptibility test

The minimum inhibitory concentration of the test agent was determined for all test *P. aeruginosa* clinical isolates as described by the Clinical and Laboratory Standards Institute, CLSI 2023^16^. Briefly, Cation Adjusted Muller-Hinton Broth (CAMHB, Oxoid) was transferred to the microtiter plate wells. Serial dilutions of OligoF were performed, and an overnight subculture of each *P. aeruginosa* test isolate was prepared using CAMHB, then diluted to get a final inoculum of 2 × 10^5^ CFU/mL. Following incubation at 35 ± 2 °C for 16–20 h, measurement of the absorbance was carried out using a microplate reader at 620 nm. The minimum inhibitory concentration (MIC) was determined as the lowest concentration of OligoF that effectively inhibited the growth of *P. aeruginosa* isolates in comparison to the control group. Furthermore, the minimum bactericidal concentration (MBC) was assessed by subculturing the entire volume from wells exhibiting no visible growth onto CAMHB plates, followed by incubation under the previously described conditions. The MBC was defined as the lowest concentration of OligoF that resulted in either a complete absence of growth or the presence of fewer than two colonies, corresponding to approximately 99.9% eradication of the initial inoculum. Furthermore, susceptibility testing of ciprofloxacin and cefepime was also done, representing standard drugs.

### Effect of OligoF on the growth curve

OligoF was diluted using LB broth to prepare concentrations of 0, ¼ MIC, or ½ MIC. An overnight culture of *P. aeruginosa* test organism was added to each test concentration to obtain a final concentration of 5 × 10^5^ CFU/mL. After incubation at 37 °C for 24 h, about 200 µl of the test *P. aeruginosa* bacterial suspension was transferred to a microtiter plate at 0, 2, 4, 6, 8, 10, 18, 24 h. Moreover, absorbance was determined at 620 nm^17^.

### Preliminary screening for biofilm-forming strains

The crystal violet method was followed to determine biofilm-forming *P. aeruginosa* isolates as designed by Kamali et al.^2^. In brief, an overnight culture of each test strain was utilized to inoculate about 5 ml of Luria Bertani (LB) broth via transferring a single colony, followed by incubation at 37 °C for 24 h under continuous shaking. Next, the culture was diluted about 200-fold by LB, then about 200 µl of each test bacterial suspension was added to the wells of a 96-well microtiter plate. After incubation of all plates under aerobic conditions at 37 °C for 18 h, gentle aspiration of all wells was performed, followed by twice washing using sterile phosphate-buffered saline (PBS, pH 7.2). Next, about 300 μl of methanol was added to each well for fixation of the test pathogen for 15 min. Next, methanol was aspirated, and all plates were left to air dry. Staining using 300 μl of 0.1% w/v crystal violet dissolved in water was carried out and left for 5 min before washing. All test plates were left to air dry again. Color intensity, which is directly proportional to the degree of biofilm formation, was assessed quantitatively using 33% (v/v) glacial acetic acid. The later could dissolve the crystal violet stain bound to adherent *P. aeruginosa* cells. Absorbance was determined at 620 nm by a Techne plate reader (Austria). This test was performed in triplicate. All test strains were classified into three groups as follows: strong-, moderate-, and weak-biofilm forming, depending on the optical density as reported by Kamali et al.^2^.

### Impact of OligoF on the colony-forming units of test isolates

A strong biofilm-forming strain was selected to induce 48 h-biofilm using 96-well microtiter plates. After that, planktonic bacterial cells were discarded, leaving adherent cells forming the biofilm, which were washed twice with PBS. Next, 200 μL of tryptic soy (TS) broth with OligoF at concentrations 0, ¼ MIC, or ½ MIC were transferred to the corresponding wells and then incubated for 24 h at 37◦C. A viable number of cells (CFU; colony forming units) was determined within pre-established biofilms by scraping and resuspension in PBS followed by vortexing. After that, test bacterial suspensions were serially diluted and cultured on MHA. All test plates were incubated for 24 h at 37 °C. Moreover, CFU was determined, and each experiment was repeated in triplicate, with the standard deviation (SD)^18^.

### Scanning electron microscopy (SEM)

For qualitative analysis of *P. aeruginosa* biofilm in the absence or presence of OligoF (0, 16, or 32 µg/ml), scanning electron microscopy was performed. Biofilm was pre-established on glass coverslips placed within 6-well microtiter plates, which were incubated at 37 °C for 24 h. After incubation, gentle aspiration was performed, followed by the addition of a fresh sterile LB containing OligoF test concentration. After incubation at 37 °C for 24 h, planktonic cells were discarded by gentle aspiration, then wells were washed with PBS twice, and the glass coverslips were put into the fixative solution, which is composed of 2.5% (v/v) glutaraldehyde in 0.1 M cacodylate buffer, pH 7. Samples were then sent to the Electron Microscope Unit, Faculty of Medicine, Tanta University, to be processed and examined with SEM^18^.

### Gel formulation

Triethylamine, carbopol 940, propylene glycol, and methylparaben were used for gel preparation according to Sitohang et al.^19^. The OligoF was added to the prepared gel in a concentration of 3 gm/100 gm gel.

### Biological evaluation

#### Animal subjects

The current study was conducted according to the guidelines for the care and use of the laboratory animals which were approved by the Research Ethical Committee, Faculty of Pharmacy, Tanta University, Egypt (TP/RE/06/22P-0019). Thirty-six male Wistar rats with a body weight of (225–250 g) and age of 8 weeks were purchased from the National Research Center (Cairo, Egypt). Rats were fed on pellet chow (El Nasr Chemical®, Cairo, Egypt). The rats were allowed free access to water and diet under identical environmental conditions. The animals were maintained for seven days for acclimatization.

The sample size was determined using SPSS to ensure statistical power of 80% at a significance level of 0.05. Based on this power analysis, 6 rats per group were deemed sufficient to detect a significant effect size, with each group representing one treatment condition. The effect size was estimated from prior studies involving similar experimental conditions.

After the acclimatization, the rats were randomly divided into six groups (n = 6): negative control group (normal rats), positive control group 1 (rats with non-infected wound and treated with vehicle gel), and OligoF treated group I (rats with non-infected wound and treated with gel preparation containing OligoF), positive control group 2 (rats with infected wound and treated with vehicle gel), OligoF treated group II (rats with infected wound and treated with gel preparation containing OligoF), and povidone-iodine treated group (rats with infected wound and treated with gel preparation containing 10% povidone-iodine). Treatments were administered, and outcome analysis was performed with blinding to the group assignments to minimize bias. The gel preparations were applied daily for 14 days on the injury site using an elastic adhesive sterile bandage.

For wound generation, the rats were anesthetized with intraperitoneal (IP) injection of a mixture of 70 mg/kg ketamine (Alfasan®, Netherland) and 7 mg/kg xylazine (Alfasan®, Netherland), and the dorsal hair was shaved from the back of the rats, then, a circular full-thickness dermal cut (1 × 1 cm) was aseptically generated. The wound was made by the use of surgical blades^20^. For a generation of infected wounds, *P. aeruginosa* suspension was applied to cover the wound immediately after wound generation.

On postoperative days (0–3–7–10–14), the injured skin was imaged using a digital camera. The area of the wound was calculated by multiplying the highest length of the injury by the perpendicular highest width. The highest length and width of the injured area were measured by a ruler^21^. Animals were monitored daily for general health, body weight, feeding behavior, and activity. No adverse effects or abnormal behaviors were observed in any treatment group throughout the study. After 14 days, rats were decapitated, and the wounded area of the skin was harvested for further assessment. In addition, normal skin samples were collected from uninjured rats in the negative control group. The study is reported in accordance with ARRIVE guidelines ([https://arriveguidelines.org).

#### ELISA

The concentration of SIRT1 in the wounded skin in the studied rat groups was done by an ELISA kit from MyBiosource (San Diego, USA) according to the manufacturer’s instructions.

#### qRT-PCR

To investigate the mechanisms by which OligoF accelerates wound healing through the SIRT1 and autophagy pathways, gene expression analysis of SIRT1, Beclin 1, and autophagy-related gene 5 (ATG5) was performed using quantitative RT-PCR. Total RNA was extracted from the skin tissue using the RNeasy Mini Kit (Qiagen, Germany), and the RNA quality was assessed by NanoDrop and gel electrophoresis to ensure that the 260/280 nm ratio was between 1.8 and 2.0, indicative of high-quality RNA. The extracted RNA was used as a template for cDNA synthesis using the QuantiTect Reverse Transcription Kit (Qiagen, Germany) in a two-step reverse transcription process.

Quantitative PCR was carried out using QuantiTect SYBR Green PCR Kit (Qiagen®, Germany), following the manufacturer’s instructions. The primer sequences used for the target genes and housekeeping gene (β-actin) were as follows:

Beclin 1 (NM_001034117.1): Forward (5′-GCC TCT GAA ACT GGA CAC G-3′), Reverse (5′-CCT CTT CCT CCT GGC TCT CT-3′), ATG5 (NM_001014250.1): Forward (5′-CACT GGG ACT TCT GCT CCT G-3′), Reverse (5′-TTC TTC AAC CAA GCC AAA CC-3′), SIRT1 (XM_006223877.1): Forward (5′-GAC GAC GAG GGC GAG GAG-3′), Reverse (5′-ACA GGA GGT TGT CTC GGT AGC-3′), β-actin (NM_031144): Forward (5′-TCC CTG GAG AAG AGC TAT GA-3′), Reverse (5′-ATA GAG CCA CCA ATC CAC AC -3′).

The qPCR amplification was carried out using the following conditions: an initial denaturation at 95°C for 15 min, followed by 40 cycles of 95°C for 15 s, 60°C for 30 s, and 72°C for 30 s. A melting curve analysis was performed to confirm the specificity of the amplification products. The relative gene expression was normalized to β-Actin as an endogenous control using 2^*−*ΔΔCT^ equation. The PCR efficiencies for the target and housekeeping genes were determined to be between 90 and 110%, ensuring accurate quantification^22,23^.

### Histopathological examination

At the end of the current experiment, the skin tissue from animals in all groups was fixed using formaldehyde (10%) for about 12 h. The skin tissue was then soaked in paraffin then Sects. (5 μm thick) of skin were cut and stained with H&E stains. The skin sections were examined using an optical microscope CX43 (Olympus, Japan). Each parameter (i.e., epithelialization, ulceration, inflammatory infiltrate, proliferation of fibroblasts, neovascularization, granulation tissue, and deposition of collagen) was scored on a scale proposed by Erlich et al. and modified by Philips et al.^24^ in which 0: no evidence, 1: occasional evidence, 2: light scattering, and 3: abundant evidence.

### Statistical analysis

Data are expressed as the mean ± SD or SE, and SPSS version 22 was used for data analysis. The statistical comparison between the studied groups was carried out using ANOVA test followed by post-hoc Fisher’s LSD. *p* < 0.05 was considered to be statistically significant.

## Results

### Characterization of OligoF and structure elucidation

The paper chromatography screening for the monomeric composition of OligoF indicated three brown spots identified as β-D-glucose, α-L-rhamnose and D-glucuronic acid as compared to the authentic sugars used.

Stepwise hydrolysis screening of OligoF resulted in the detection of β-D-glucose after 15 min, while a mixture of β-D-glucose and D-glucuronic acid was detected after 30 min. α-L-rhamnose was not detected at this time interval. This result indicated that the terminal sugars are β-D-glucose and D-glucuronic. At the intervals 45 min to 120 min, α-L-rhamnose was detected alongside β-D-glucose and D-glucuronic acid which augments the suggestion that the proposed sequence of the sugars in OligoF is β-D-glucose- α-L-rhamnose- D-glucuronic acid.

NMR analysis supported the results of the paper chromatography screening (Table 1). ^1^H NMR and ^13^C NMR evidenced 4 anomeric protons and carbons, respectively. NMR spectra of OligoF are provided in the supporting data file.

**Table 1:** ^13^CNMR chemical shifts (ppm) of OligoF (D_2_O, 125MHz).

Sugar residue	^13^C NMR					
	C1	C2	C3	C4	C5	C6
β-D-Glu (2 →	98.5	78.5	72.1	72.2	69.1	63.2
→ 4) β-D-Glu (2 →	98.5	78.6	72.1	78.3	69.1	63.3
→ 1) α -L -Rh (4 →	97.6	76.8	74.1	76.7	68.9	21.9
→ 4) α -D-GlA	94.6	76.6	73.9	78.4	72.3	169.4

^1^H NMR spectrum exhibited doublet signals at δ_H_ 5.12, 4.84, and 4.54 integrated for one, one and two protons, respectively. The HSQC data of OligoF indicted the correlations of the δ_H_ 5.12 to the δ_C_ 94.6, the δ_H_ 4.84 to the δ_C_ 97.6 and the δ_H_ 4.54 to the δ_C_ 98.5. The DEPTQ spectrum revealed that the δ_C_ 94.6, 97.6 and 98.5 are for (C–H) carbons. Additionally, the ^13^C NMR and DEPTQ data identified a signal for (C = O) carbon at δ_C_ 169.4 which supported the inclusion of D-glucuronic acid. A signal at δ_C_ 21.9 showed correlation to δ_H_ 1.33 (integrated for 3 protons) which was assigned to the methyl group of rhamnose moiety. The coupling constants for δ_H_ 5.12, 4.84, 4.54 are 2.4, 2.6 and 6.8 Hz implying the sugar configurations are α, α , and β, respectively. By inspecting the δ_C_ for the individual sugars the signals at δ_C_ 94.6 and 98.5 matches the anomeric carbons for α-D-glucuronic acid and β-D-glucose compared to the literature^25,26^ while the β-D-glucuronic acid and α -D-glucose anomeric carbons are reported in the literature at 98.2 and 90. The signal δ_H_ 4.84 –– δ_C_ 97.6 was assigned to the anomeric position of rhamnose as reported in the published literature for C-1 linked rhamnose^27^. These chemical shifts indicated that only the anomeric positions of rhamnose is included in the glucosidic linkages. The signals at δ_C_ 63.2 and 63.3 for (–CH_2_) were assigned to C-6 of two glucose sugars indicating that C-6 is not included in the glucosidic linkage^28^. The signals at δ_C_ 78.6 and 78.5 were assigned for C-2 for the two glucose units. The chemical shift for the latter carbons matches the downfield shift effect of the incorporation of this site in a glucosidic linkage^28^. Thus, the glucose units are linked through C-2. For glucuoronic acid, the signal at δ_C_ 78.4 was assigned to the C-4 position of glucuronic acid, while the δ_C_ 78.3 was attributed to C-4 of one of the glucose units; these chemical shifts are downfield by ~ 4.1 ppm, which could be due to the inclusion of these positions in the glucosidic linkage^25,28^. For the rhamnose unit, the signals at δ_C_ 76.8 and 76.7 were assigned to the C-2 and C-4 positions; these chemical shifts are downfield by ~ 6.0 ppm compared to the literature^27,28^, which could be due to its inclusion in the glucosidic linkage. Thus, our data propose a linear tetra-saccharide composed of β-D-glucose as one terminal end and is linked through C-2, β-D-glucose as an internal sugar and linked through C-2 and C-4, α-L-rhamnose as an internal sugar linked through C-1 and C-4, and α -D-glucuronic acid as the other terminal end linked through C-4.

To determine the sugar sequence, the NOESY spectrum was inspected. The triplet of H-5 of one glucose unit at δ_H_ 3.1 was correlated to the anomeric proton of glucose at δ_H_ 4.54. Moreover, the anomeric proton of rhamnose was correlated to the anomeric proton for glucose unit, at δ_H_ 4.54. These correlations arranged the sugars in the order β-D-glucose (C2 → C-4) β-D-glucose (2 → 1) α -rhamnose (4 → 4) α -D-glucuronic acid.

### Antibacterial activity of OligoF

**Fig. 1:**
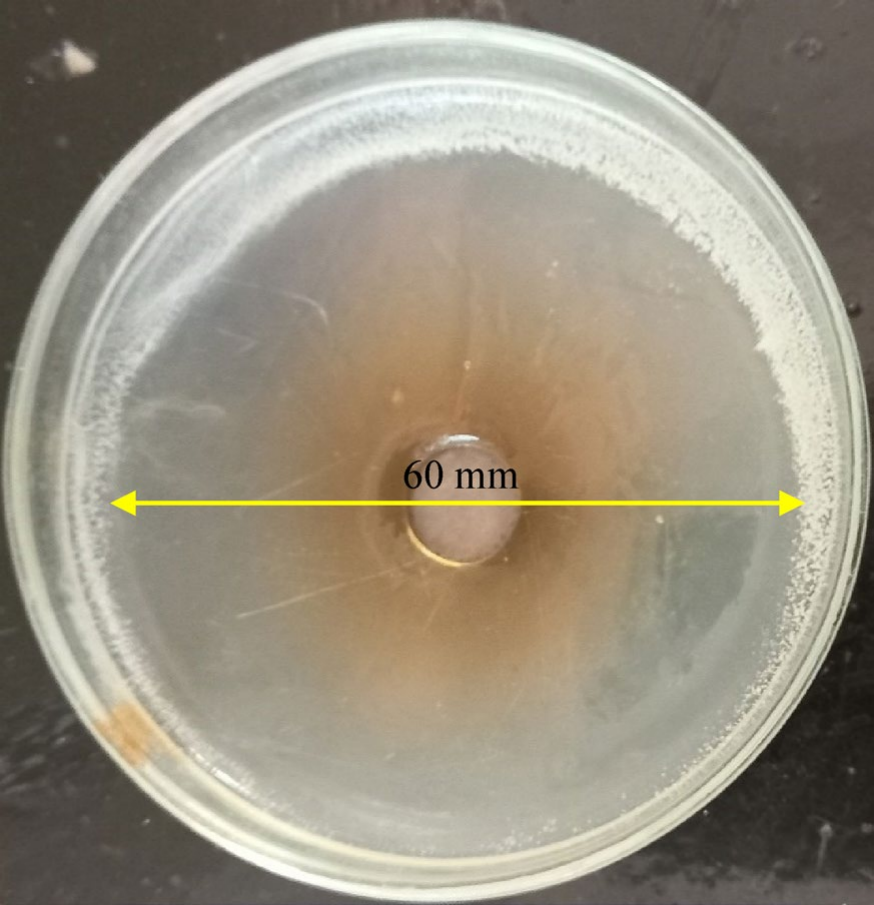
Agar well diffusion test showing the susceptibility of an MDR *Pseudomonas aruginosa* isolate to OligoF, recording a growth inhibition zone of 60 mm.

Preliminary screening for antibacterial activity of OligoF against MDR *P. aeruginosa* isolates revealed large inhibition zone diameters ranging between 60–63 mm as shown in Fig. 1 and also recorded in Table 2. Additionally, MIC (16 µg/ml) and MBC (32 µg/ml) values were also recorded using broth microdilution assay (Table 2). Also, the susceptibility of the test isolates to ciprofloxacin and cefepime was presented in Table 2.

**Table 2:** Assessment of *P. aeruginosa* growth inhibition by OligoF using agar well diffusion method in addition to the determination of the minimum inhibitory concentration (MIC) as well as the minimum bactericidal concentration (MBC) in µg/mL of OligoF against the test isolates.

Isolate code	Inhibition zone diameters against test isolates (mm)*	MIC and MFC of OligoF (µg/mL)		MIC and MFC of ciprofloxacin (µg/mL)**		MIC and MFC of cefepime (µg/mL)**	
		MIC	MFC	MIC	MFC	MIC	MFC
P1	60 ± 1.15	16	32	4	16	32	64
P2	60 ± 1.15	16	32	4	8	64	128
P3	60 ± 1.15	16	32	4	8	64	128
P4	63 ± 0.57	16	32	4	8	64	128
P5	60 ± 1.15	16	32	4	16	32	64
P6	62 ± 0.57	16	32	4	8	32	64
P7	60 ± 1.15	16	32	4	8	64	128
P8	60 ± 1.15	16	32	4	8	32	64
P9	62 ± 0.57	16	32	8	8	64	128
P10	60 ± 1.15	16	32	4	8	32	64
*P. aeruginosa* ATCC 27,853	65 ± 0.57	0.25	0.5	0.25	0.5	0.25	0.5

* Mean of the diameter and standard deviation. Data represented three trials.

** Break points of ciprofloxacin and cefepime against *P. aeruginosa* are ≥ 4 and ≥ 32, respectively.

**Fig. 2:**
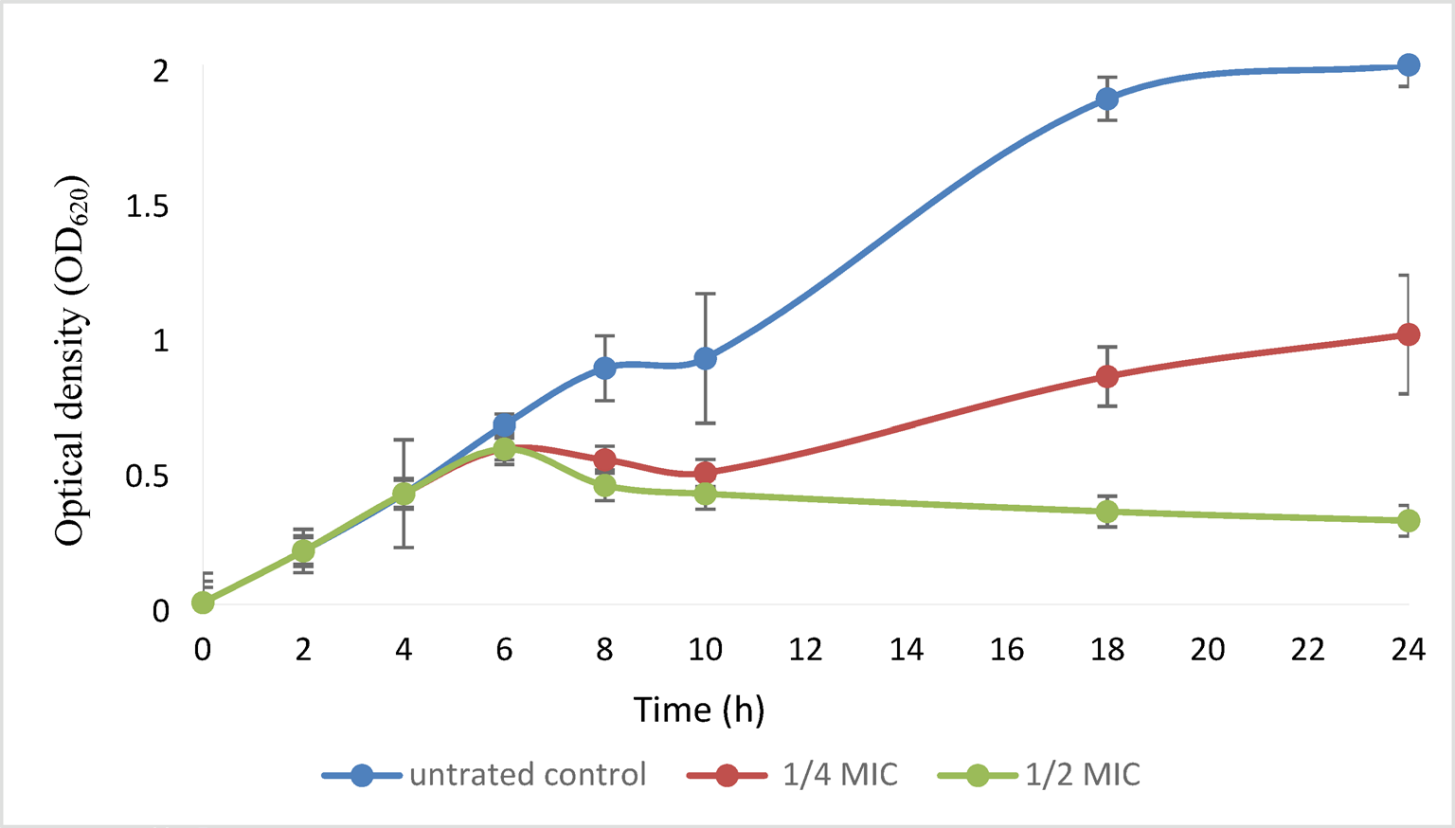
Growth curve of *P. aeruginosa* isolate in the absence and presence of sub-MIC concentrations of OligoF showing a concentration-dependent reduction in the optical density of treated cells compared to the untreated control. Three independent repeated trials were performed.

The inhibitory activity of OligoF on the growth of *P. aeruginosa* isolate was shown in Fig. 2. Adding either of ¼ or ½ MIC of the test agent was associated with a temporary growth increase in the OD_620_ for the next 6 h, followed by a continuous reduction treatment by ½ MIC until 24 h exerting ~ 1.7-fold reduction compared to the control.

**Fig. 3:**
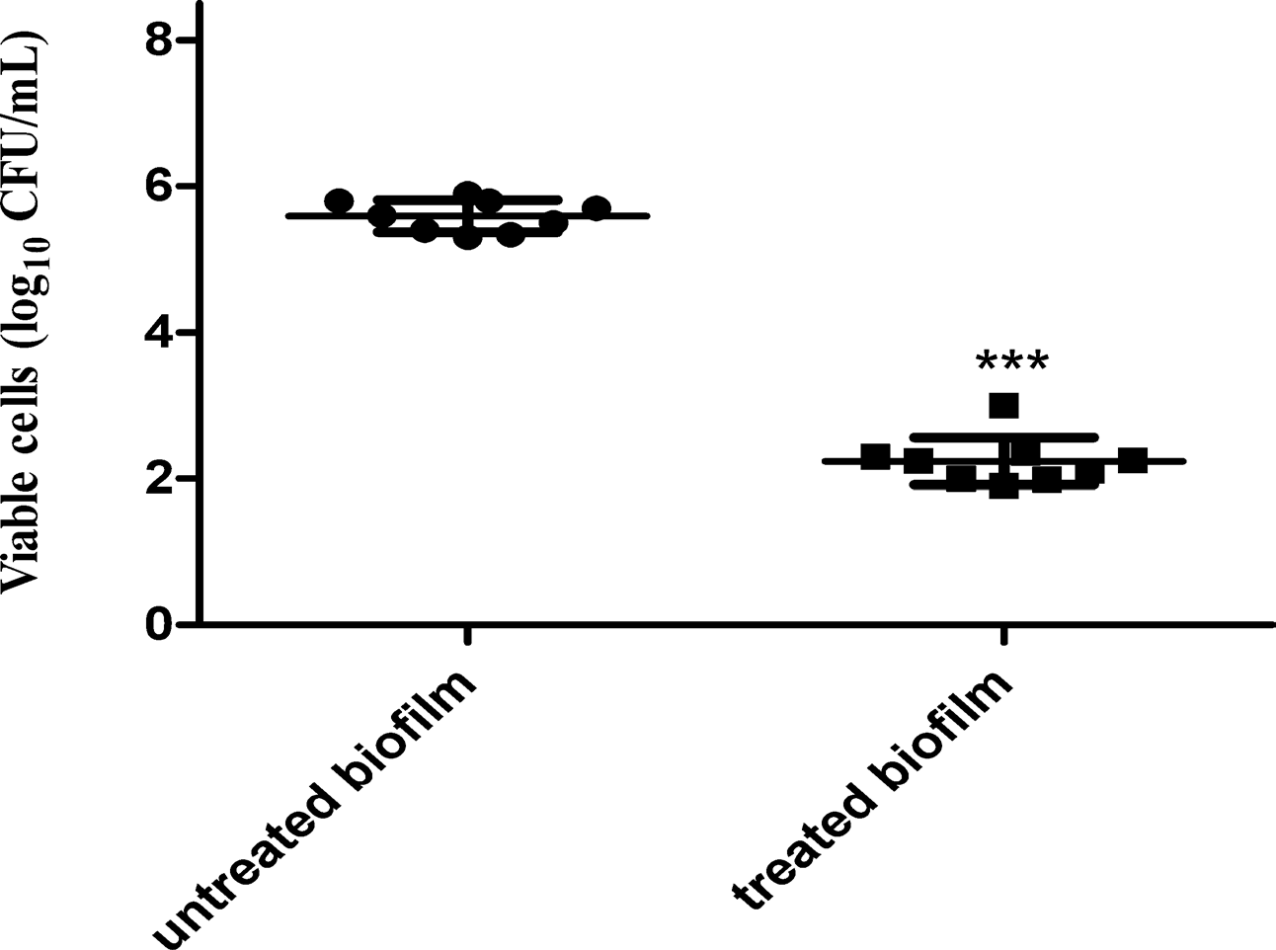
Impact of ½ MIC of OligoF on the viable cell count of *P. aeruginosa* pre-established biofilm, showing dramatic reductions in the treated biofilm compared to the control. Error bars denote standard deviations (SD) and asterisks indicate statistical significance (*** *p* < 0.001).

The effect of ½ MIC of OligoF on the viability of *P. aeruginosa* cells in a pre-established biofilm revealed significant reductions (~ fourfold) in the treated biofilm compared to the untreated control (Fig. 3).

**Fig. 4:**
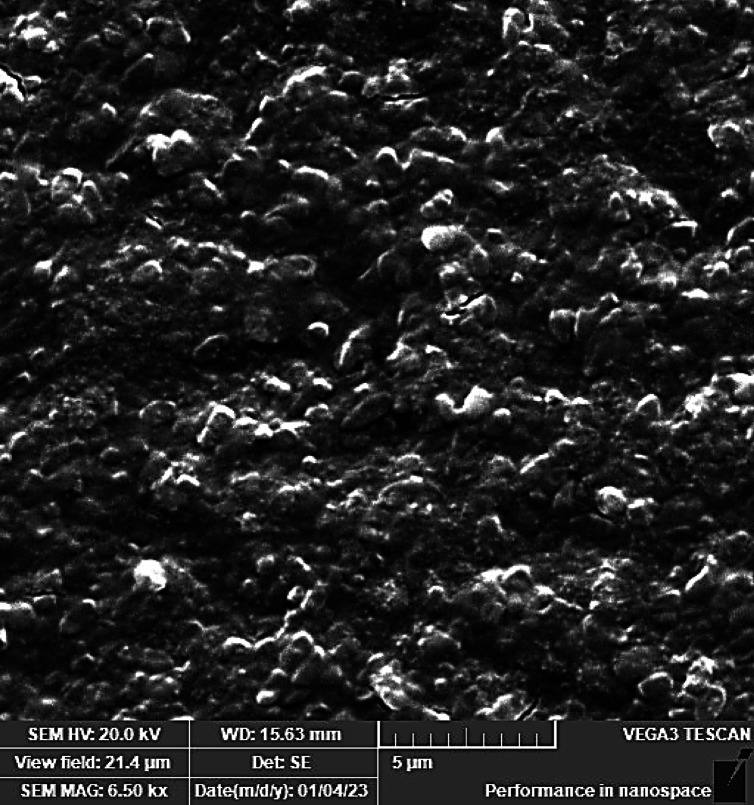
Micrographs of scanning electron microscope displaying untreated *P. aruginosa* biofilm showing intact pathogenic cells in huge amounts covered with a thick matrix of extracellular polysaccharides.

**Fig. 5:**
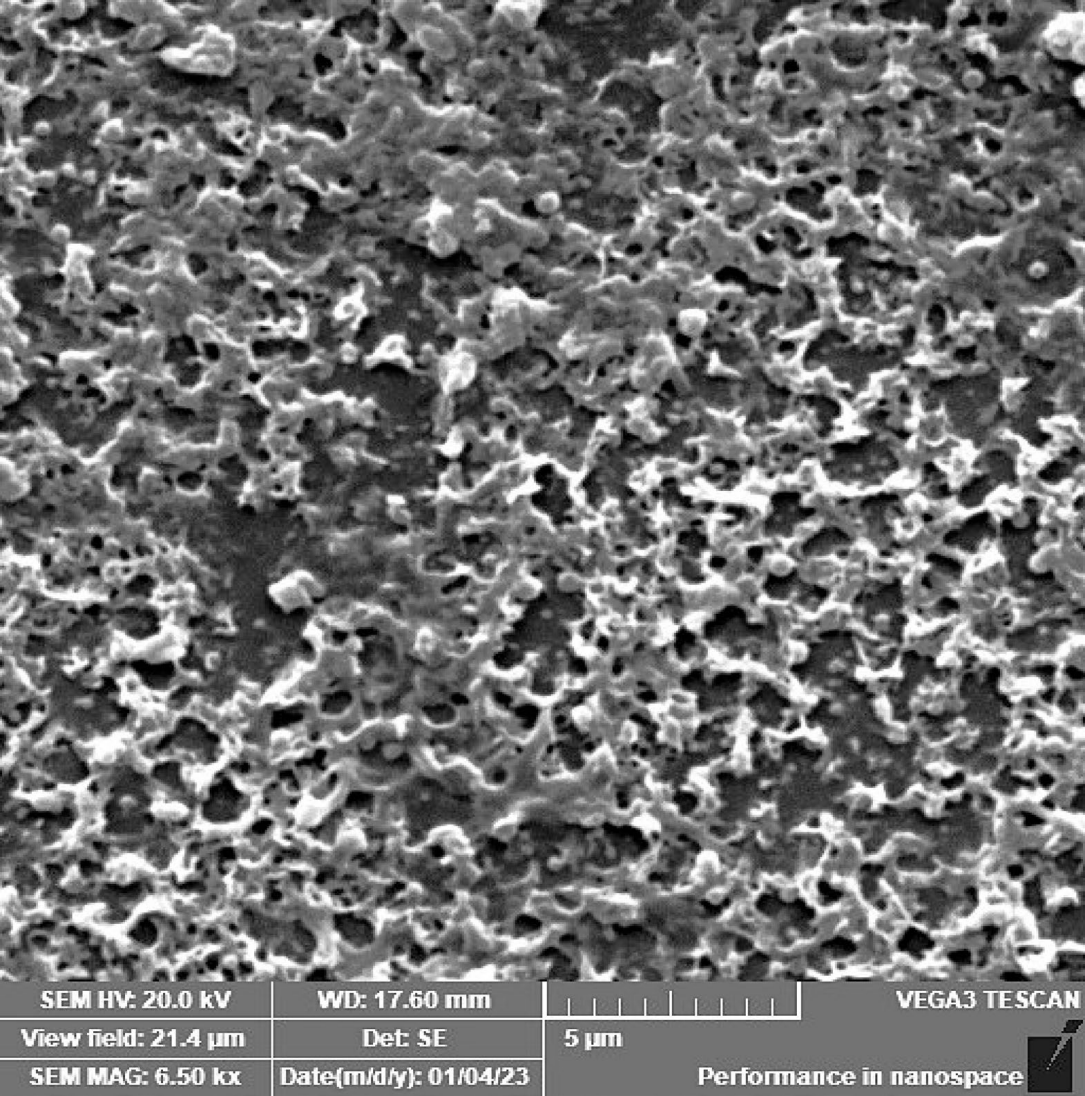
Micrographs of scanning electron microscope displaying pre-established biofilm showing cellular distortion following treatment with 8 µg/mL of OligoF.

**Fig. 6:**
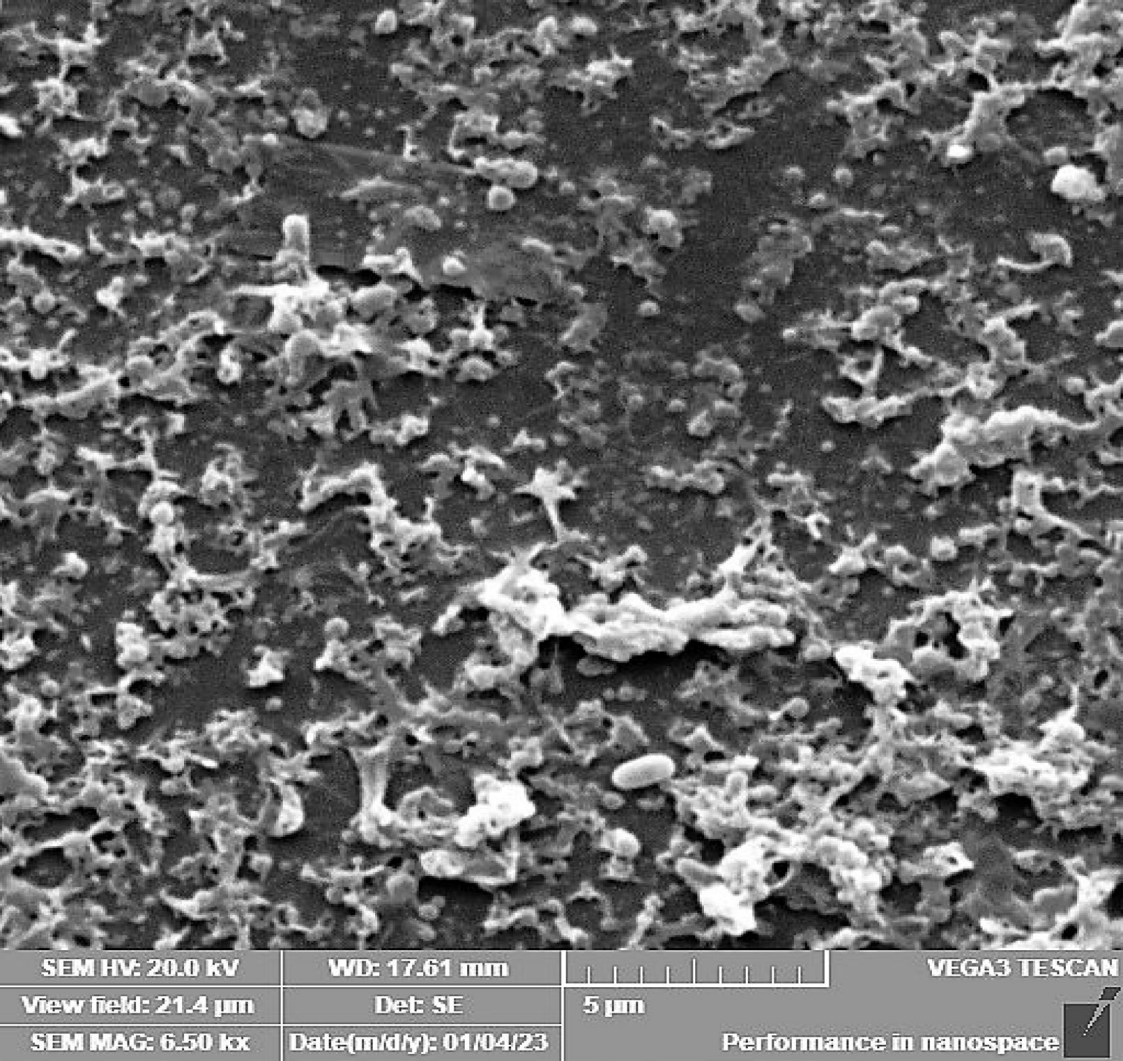
Micrographs of scanning electron microscope displaying pre-established biofilm showing large spaces between cells in addition to severe damage and cellular distortion following treatment with 32 µg/mL of OligoF.

Results of scanning electron microscopy displayed a preformed biofilm of untreated *P. aeruginosa* showing intact pathogenic cells in huge amounts covered with a matrix of extracellular polysaccharides (Fig. 4). On the other hand, large spaces between cells beside cellular distortion following treatment with 8 µg/mL (static concentration) of OligoF (Fig. 5). Additionally, more wide spaces between the bacterial cells in addition to severe damage and cellular distortion following treatment with 32 µg/mL (cidal concentration) of OligoF (Fig. 6).

### Biological evaluation

#### Effect of OligoF on wound’s area

Treatment of non-infected wound with OligoF showed a significant reduction in the wound area compared to untreated wound in the positive control group 1 (rats with non-infected wound) at days 3, 7, 10, and 14 postoperatively (*p* < 0.001). Moreover, treatment of infected wound with OligoF or povidone-iodine showed a significant reduction in wound area compared to untreated infected wound in the positive control group 2 at days 3, 7, 10, and 14 postoperatively (*p* < 0.001).

**Fig. 7:**
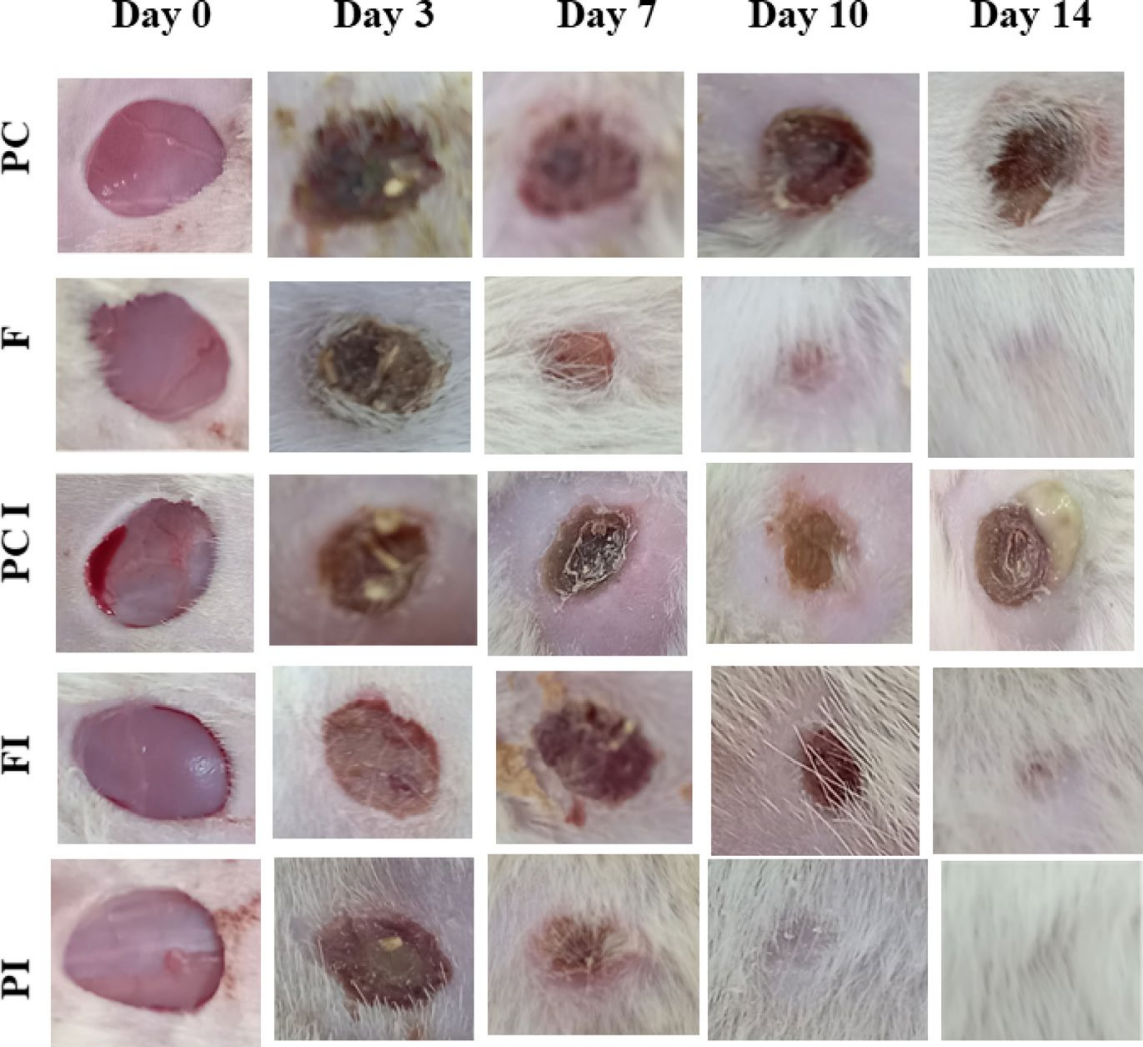
Representative images of wounded areas in the dorsal side of rats in the studied groups throughout the experiment (at days 0, 3, 7, 10, and 14 after injury). PC: Rats with non-infected wound (positive control 1), F: Rats with non-infected wound and treated with OligoF, PCI: Rats with infected wound (positive control 2), FI: Rats with infected wound and treated with OligoF, and Betadine PI: Rats with infected wound and treated with Povidone-iodine.

**Fig. 8:**
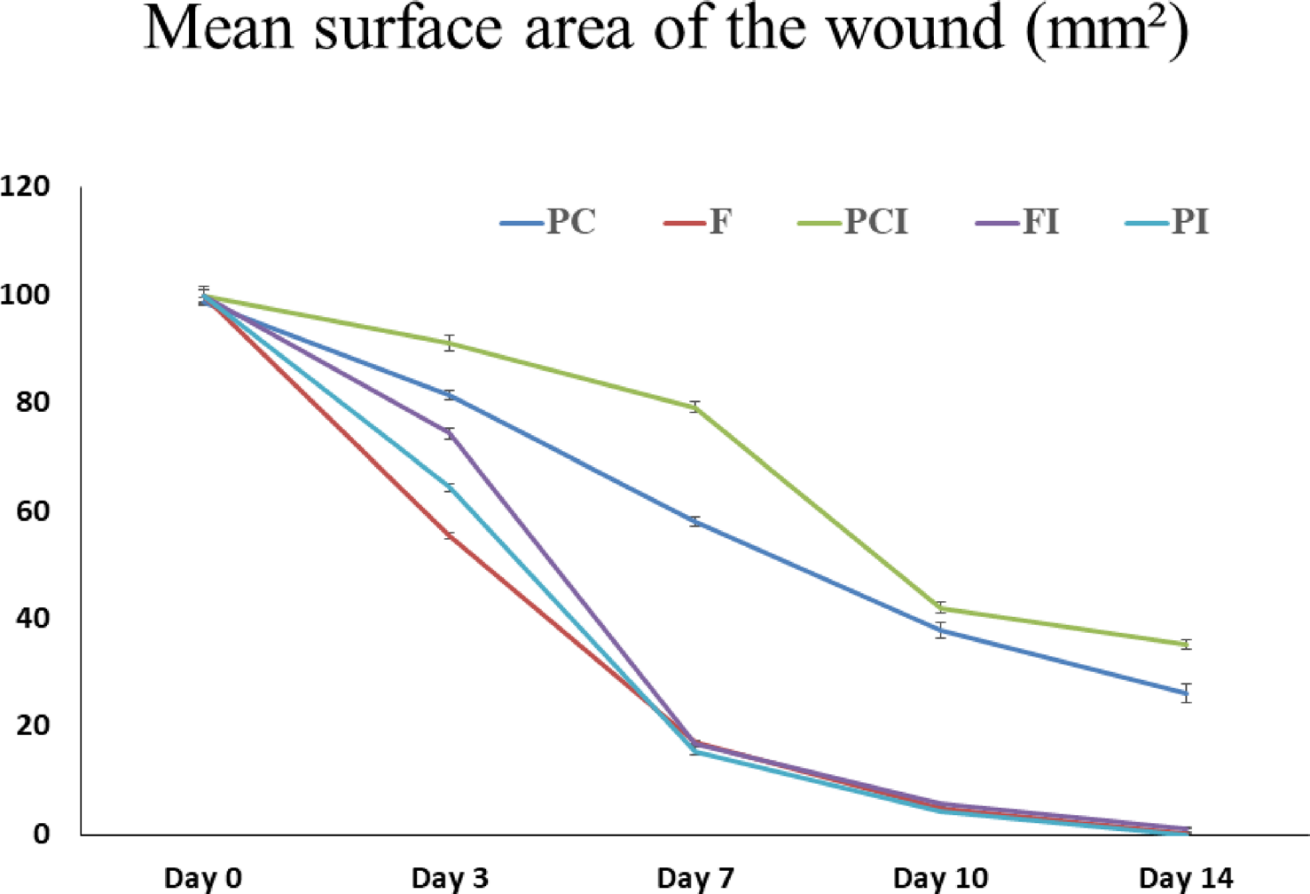
The wounded area calculated among the studied animal groups throughout the experiment (at days 0, 3, 7, 10, and 14 after injury). *n* = 6, data are presented as mean ± SE. PC, Rats with non-infected wound (positive control 1), F: Rats with non-infected wound and treated with OligoF, PCI: Rats with infected wound (positive control 2), FI: Rats with infected wound and treated with OligoF, and PI: Rats with infected wound and treated with Povidone iodine.

In addition, infected wound treated with povidone-iodine showed a significant reduction (*p* < 0.001) in wound area compared to infected wound treated with OligoF at day 3 postoperatively (Table 3) (Figs. 7 and 8). More interestingly, the application of OligoF or povidone-iodine on infected wound areas obviously decreased signs of inflammation and the presence of green discharge in infected wound areas (Fig. 7). Throughout the experimental period, no treatment-related side effects were observed in animals treated with OligoF or povidone-iodine. Body weight, feeding, and activity remained normal in all groups.

**Table 3:** Effect of topical application different treatments on the surface area of the wound (mm^2^).

Group	Day 0	Day 3	Day 7	Day 10	Day 14
PC	98.67 ± 0.76	81.25 ± 0.87^b,c,d^	58.08 ± 0.86^b,c,d^	37.79 ± 1.55^b,c,d^	26.17 ± 1.82^a,b,d^
F	99.67 ± 1.31	55.50 ± 0.60^a,c,d^	17.08 ± 0.35^a,c^	4.83 ± 0.31^a,c^	0.33 ± 0.21^a,c^
PCI	99.83 ± 1.30	90.92 ± 1.47^a,b,d^	79.17 ± 0.86^a,b,d^	42.00 ± 1.10^a,b,d^	35.13 ± 0.90^a,b,d^
FI	99.83 ± 1.83	74.29 ± 0.99^a,b,c^	16.92 ± 0.42^a,c^	5.67 ± 0.21^a,c^	1.17 ± 0.11^a,c^
PI	99.67 ± 1.31	64.33 ± 0.76^a,b,c,d^	15.50 ± 0.71^a,c^	4.33 ± 0.21^a,c^	0 ± 0^a,c^

*N* = 6, *p* = 0.05.

a, significant versus PC.

b, significant versus F.

c, significant versus PCI.

d, significant versus FI.

PC, Rats with non-infected wound (positive control 1).

F, Rats with non-infected wound and treated with compound OligoF.

PCI, Rats with infected wound (positive control 2).

FI, Rats with infected wound and treated with compound OligoF.

PI, Rats with infected wound and treated with Povidone iodine.

#### Effect of OligoF on the concentration of SIRT1 in wounded skin

**Fig. 9:**
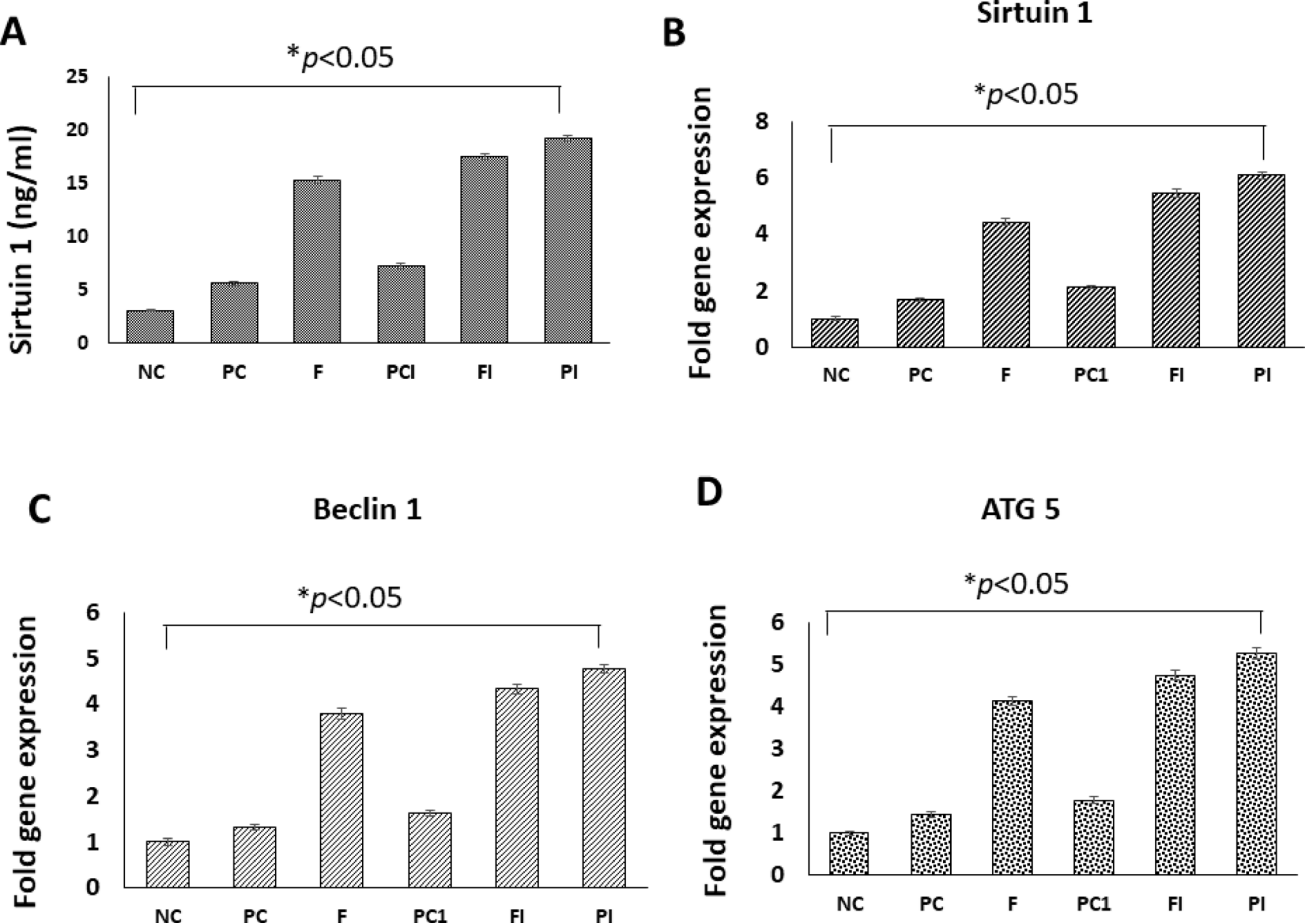
Effect of topical application of different treatments on sirtuin 1 concentration (**A**) and gene expression of *SIRT1* (**B**), *beclin 1* (**C**), and ATG5 (**D**) in wounded skin. * Significant difference from other groups (*p* < 0.05).

At the end of our experiment, SIRT1 concentration in wounded area was measured using the ELISA technique. Figure 9A shows that treatment of a non-infected wound with OligoF significantly increased SIRT1 concentration in the wounded area compared to untreated wound in the positive control group 1 (*p* < 0.001). Moreover, treatment of infected wound with OligoF or povidone-iodine significantly increased SIRT1 concentration in wounded area compared to untreated infected wound in the positive control group 2 (*p* < 0.001). In addition, the concentration of SIRT1 in infected wound treated with povidone iodine was significantly higher than its concentration in infected wound treated with OligoF (*p* < 0.001).

#### Effect of OligoF on the gene expression of SIRT1, beclin 1, and ATG5 in wounded skin

At the end of our experiment, the gene expression of SIRT1 and autophagy markers (*Beclin 1–ATG5*) in wounded area were determined using real-time PCR. Our data showed that, treatment of non-infected wound with OligoF significantly increased the gene expression of *SIRT1* (4.43 ± 0.11 fold), *beclin 1* (3.80 ± 0.13 fold), and *ATG5* (4.13 ± 0.11 fold) in wounded area compared to untreated wound in the positive control group 1 (1.67 ± 0.05, 1.32 ± 0.06, and 1.44 ± 0.07 fold, respectively, *p* < 0.0001).

Moreover, treatment of infected wound with OligoF or povidone iodine significantly increased the gene expression of SIRT1 (5.45 ± 0.15 and 6.09 ± 0.13 fold, respectively), *beclin 1* (4.34 ± 0.11 and 4.78 ± 0.10 fold, respectively), and ATG5 (4.75 ± 0.12 and 5.27 ± 0.14 fold, respectively) in the wounded area compared to untreated infected wound in the positive control group 2 (2.12 ± 0.07, 1.62 ± 0.07, and 1.78 ± 0.08 fold, respectively* p* < 0.0001). In addition, the gene expression of *SIRT1, beclin 1*, and *ATG5* in infected wound treated with povidone-iodine were significantly higher than their expression in infected wound treated with OligoF (*p* < 0.05) (Fig. 9B, C, and D).

This comparison indicates that while both OligoF and povidone-iodine enhance SIRT1 and autophagy-related gene expression, povidone-iodine appears to induce a slightly stronger upregulation in infected wounds, whereas OligoF may provide additional host repair benefits in non-infected wounds through the activation of the SIRT1–autophagy axis. These differences suggest that OligoF and povidone-iodine may promote wound healing via distinct mechanisms.

#### Histopathological examinations

**Fig. 10:**
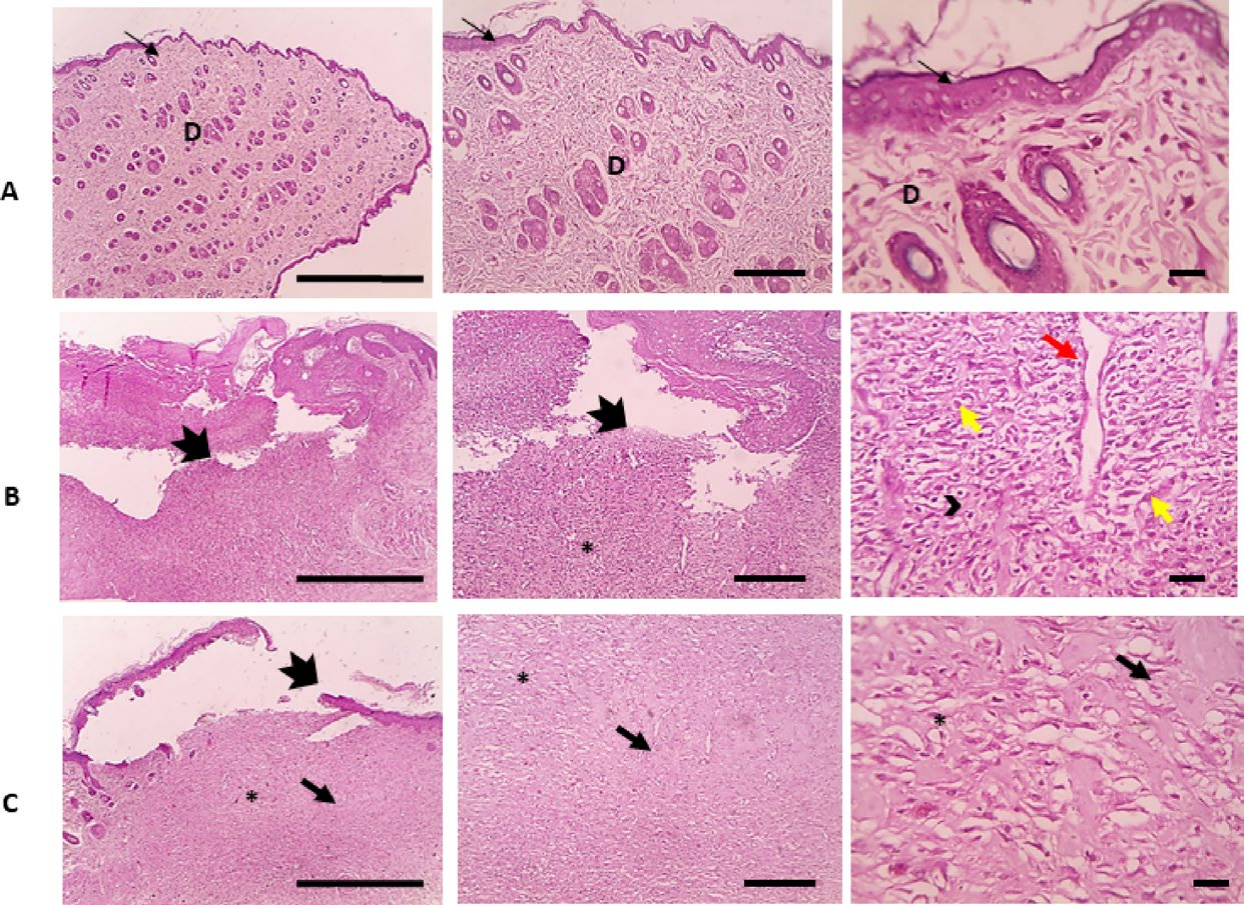
Microscopic images of HE-stained skin sections of (**A**) normal skin in the negative control group showing normal epidermis (thinning arrows) and dermis (**D**) containing normal sweat glands, sebaceous glands and, hair follicles, (**B**) wounded skin in the control positive group 1 (PC) showing obvious loss of epidermal layers which replaced by heavy polymorphonuclear cells infiltration (dense arrows) and granulation tissue filling the wound gap (*) composed of young capillary beds (red arrows), mononuclear cells (headed arrow), and immature fibroblasts (yellow arrows) infiltration, and (**C**) wounded skin treated with OligoF showing separation of new epidermis (thick arrow) with granulation tissue (*) and little amount of vascularized connective tissue deposition (thin black arrow) filling wound gap. (Magnifications: left column × 40 bar 200, mid column × 100 bar 100, and right column × 400 bar 50).

At the end of the current experimental study, the animals were sacrificed and the skin from the injured area was collected for histopathological examination. The microscopic examination of HE-stained skin sections from normal skin in the negative control group showed a normal epidermis and dermis containing normal sweat glands, sebaceous glands, and hair follicles (Fig. 10A). On the other hand, wounded skin in the control positive group 1 (PC) showed obvious loss of epidermal layers which was replaced by heavy polymorphonuclear cells infiltration and granulation tissue filling the wound gap composed of young capillary beds, mononuclear cells, and immature fibroblasts infiltration (Fig. 10B). Moreover, a microscopic examination of wounded skin treated with OligoF showed separation of new epidermis with granulation tissue and little amount of vascularized connective tissue deposition filling wound gap (Fig. 10C).

**Fig. 11:**
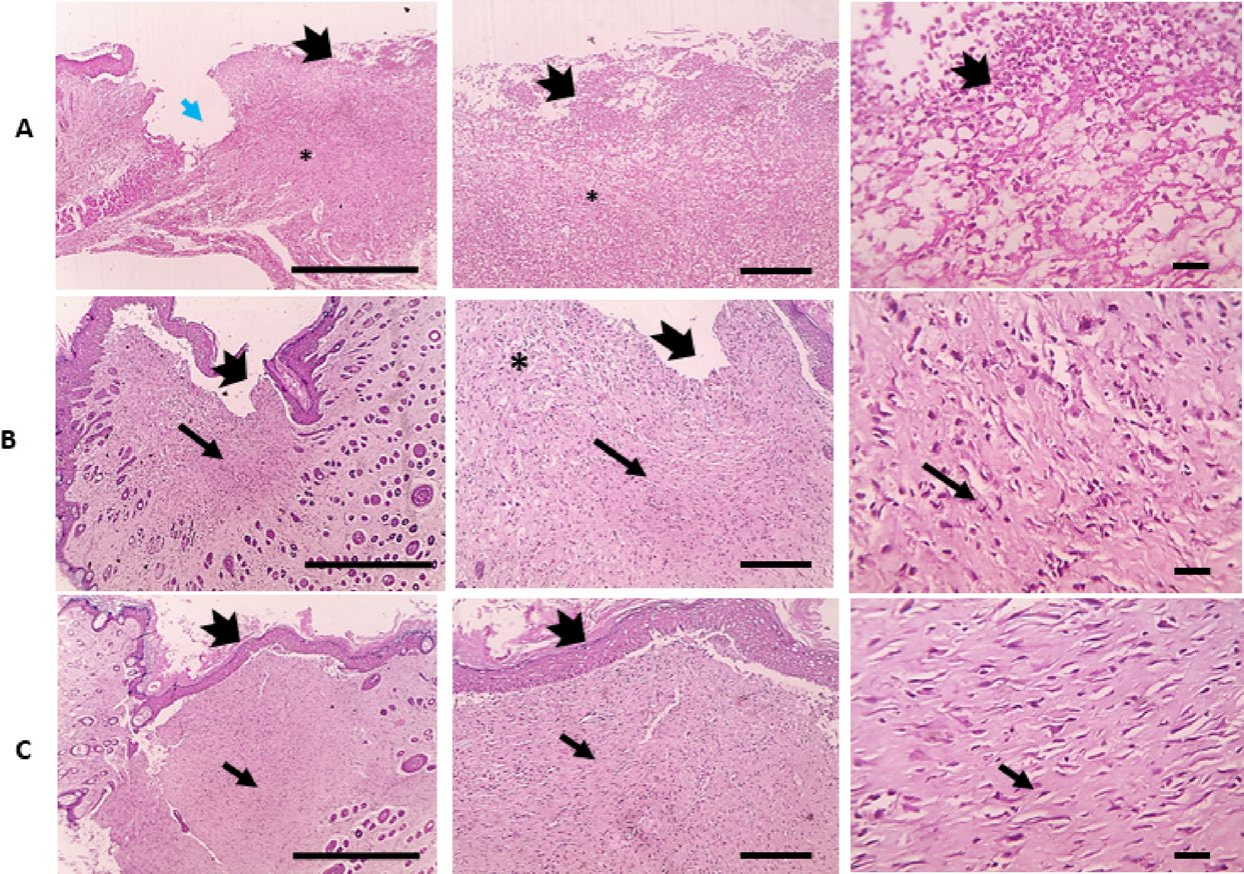
Microscopic images of HE-stained skin sections from (**A**) infected wounded skin in the positive control group 2 (PCI) showing ulceration (blue arrow), loss of epidermal layers that replaced by intense polymorphonuclear cells infiltration (thick arrows) with granulation tissue filling wound gap (*), (**B**) infected wound treated with OligoF showing separation of new epidermis (thick arrow) with little amount of granulation tissue and higher amount of vascularized connective tissue deposition (thin black arrow) filling wound gap than in infected group (PCI), and (**C**) infected wound treated with povidone-iodine showing completed re-epithelization (thick arrow) with slightly separated new epidermis, and full amount of well-organized mature connective tissue deposition (thin black arrow) filling wound gap. The contraction of wound gap is obvious in this group. (Magnifications: left column × 40 bar 200, mid column × 100 bar 100 and right column × 400 bar 50).

More interestingly, microscopic examination of HE-stained skin sections from infected wounded skin in the positive control group 2 (PCI) showed ulceration and loss of epidermal layers that were replaced by intense polymorphonuclear cells infiltration with granulation tissue filling wound gap (Fig. 11A). On the other hand, infected wound treated with OligoF showed separation of the new epidermis with a little amount of granulation tissue and a higher amount of vascularized connective tissue deposition filling wound gaps than in the infected group (PCI) (Fig. 11B). In addition, infected wound treated with povidone-iodine showed completed re-epithelization with slightly separated new epidermis and a full amount of well-organized mature connective tissue deposition filling wound gaps with obvious contraction of wound gap (Table 4) (Fig. 11C).

**Table 4:** Score of ulceration, epithelialization, inflammatory infiltrate, proliferation of fibroblasts, granulation tissue, deposition of collagen, and neovascularization.

Scores	PC	F	PCI	FI	PI
Ulceration	3 3 3	0 0 0	3 3 3	2 1 1	0 0 0
Epithelialization	0 0 0	3 3 3	0 0 0	2 1 1	3 3 3
Inflammatory infiltrate	3 3 3	1 0 1	3 3 3	1 1 1	1 0 0
Proliferation of fibroblasts	2 2 2	3 3 3	2 2 2	2 3 2	3 3 3
Neovascularization	2 1 2	2 2 1	2 2 3	2 2 2	1 1 2
Deposition of collagen	1 0 0	3 3 3	0 0 0	2 1 1	3 3 3
Granulation tissue	3 3 3	0 0 0	3 3 3	2 1 2	0 0 0

PC, Rats with non-infected wound (positive control 1); F, Rats with non-infected wound and treated with compound OligoF; PCI, Rats with infected wound (positive control 2); FI, Rats with infected wound and treated with compound OligoF; PI, Rats with infected wound and treated with Povidone iodine.

**Fig. 12:**
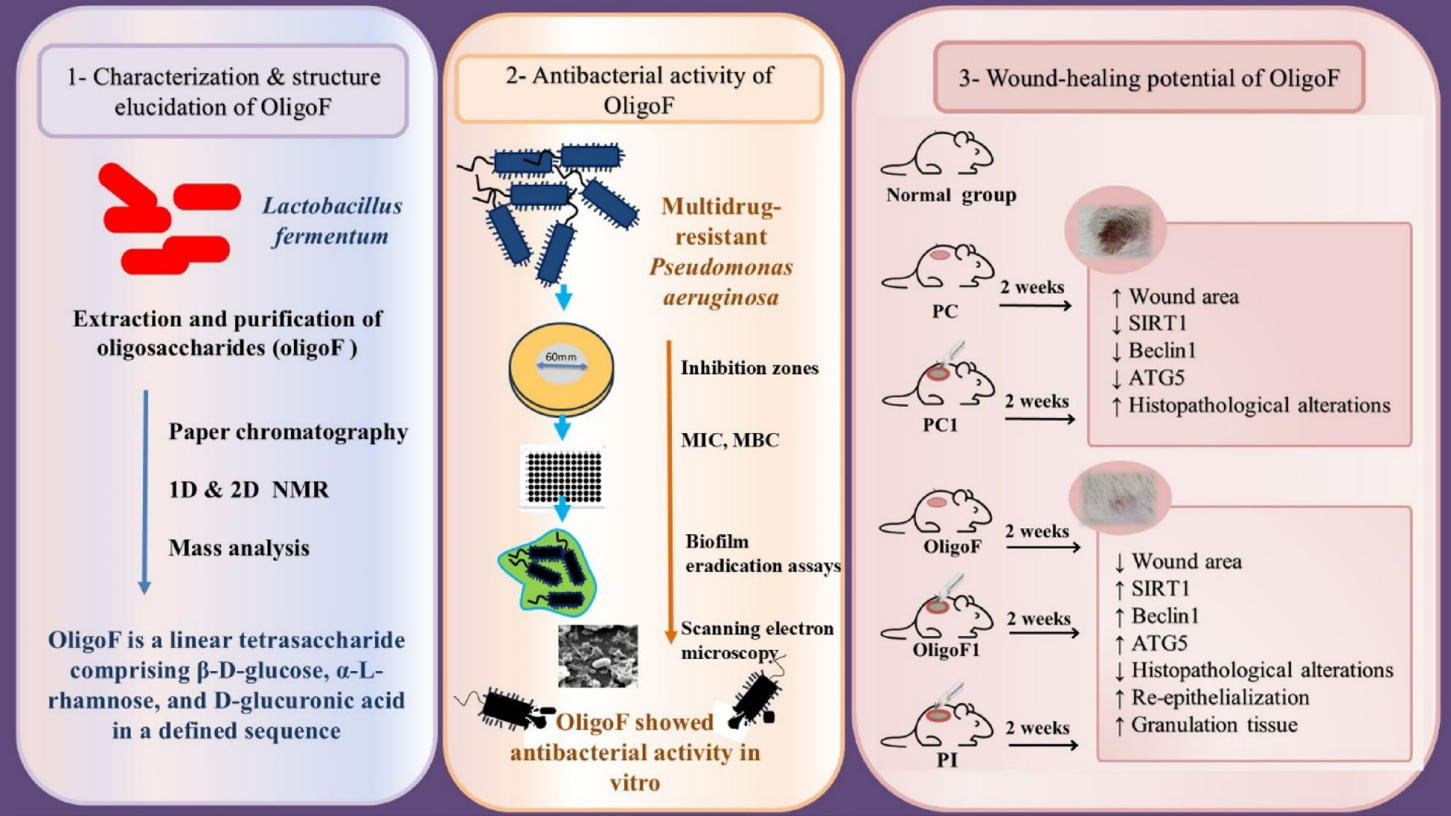
Graphical summary showing characterization of Oligof and its dual role in antibacterial action and wound repair.

A graphical summary illustrates the characterization of Oligof and its dual role in antibacterial activity and wound healing (Fig. 12). The figure highlights the key physicochemical features of Oligof, its mechanism of disrupting bacterial integrity, and its ability to promote tissue repair. Together, these findings underscore Oligof’s therapeutic potential in antibacterial, antibiofilm, and wound-repair applications.

## Discussion

Biofilm is considered a type of *P. aeruginosa* virulence that could lead to bacterial resistance, hence making this pathogen harder to eradicate. Furthermore, this community of bacterial cells is sheathed within an extracellular polymeric matrix including exopolysaccharides as essential components of this matrix^1^.

Strategies of anti-biofilm involved several approaches like aggressive antibiotic treatment, anti-adhesive agents, and high-dose antimicrobial combinations. Moreover, anti-biofilm drugs have two main targets including bacterial cells as well as the complex matrix^29^. In the present study, OligoF demonstrated robust antibacterial activity against planktonic multidrug-resistant *P. aeruginosa*, as reflected by inhibition zones of 60–63 mm, an MIC of 16 µg/mL, and an MBC of 32 µg/mL. For comparison, ciprofloxacin and cefepime typically display lower MICs against susceptible *P. aeruginosa* isolates, with reported ranges of 0.5–64 µg/mL for ciprofloxacin and MIC₉₀ values around 8 µg/mL for cefepime; however, their efficacy diminishes significantly in the biofilm state due to impaired penetration and reduced bacterial metabolic activity^30^. Cefepime, in particular, has been reported to face resistance rates exceeding 70% in *A. baumannii* and ~ 25% in *P. aeruginosa*, with non-susceptibility strongly correlated with enhanced biofilm formation^31^. In contrast, OligoF retained substantial antibiofilm potency, achieving a ~ fourfold reduction in biofilm viability at sub-MIC concentrations. SEM analyses provided further confirmation: untreated *P. aeruginosa* biofilms displayed intact, densely packed communities, while OligoF exposure at 8 µg/mL (static concentration) induced clear enlargement of intercellular spaces, accompanied by cellular distortion. At 32 µg/mL (cidal concentration), OligoF produced even more pronounced effects, with wide gaps between bacterial cells and severe structural damage, consistent with previously reported observations^11,12,32^. Such marked matrix disruption and cellular distortion contrast with ciprofloxacin or cefepime treatments, which are frequently associated with partial biofilm survival and preserved architecture. Taken together, these findings indicate that OligoF exerts potent effects against both planktonic and biofilm-associated *P. aeruginosa*, overcoming limitations commonly observed with standard antibiotics and supporting its promise as a novel therapeutic option.

Wound healing is a fundamental physiological process that is utilized to maintain the integrity and function of the skin. The impairment of wound healing process presents a major problem worldwide^3^. The present study aimed to assess the effects of OligoF on wound healing process by comparing the effect of OligoF with povidone-iodine (10%) on the healing of infected wound and try to investigate the underlying mechanism of action. Our study showed that, treatment of non-infected wound with OligoF showed a significant reduction (*p* < 0.001) in the wound area compared to untreated wounds in the positive control group 1 from days three postoperatively. Moreover, treatment of infected wound with OligoF or povidone-iodine showed also a significant reduction (*p* < 0.001) in wound area compared to untreated infected wound in the positive control group 2 from days three postoperatively. More interestingly, infected wound areas treated with OligoF showed no significant difference in infected wound areas treated with povidone-iodine from day seven postoperatively and application of OligoF or povidone-iodine on infected wound areas obviously decreased signs of inflammation and the presence of green discharge in infected wound area.

Our data obtained from observation and calculation of injured surface area were in agreement with our histopathological findings. The microscopic examination of wounded skin treated with OligoF showed separation of the new epidermis with granulation tissue and little amount of vascularized connective tissue deposition filling the wound gap. On the other hand, infected wound treated with OligoF showed separation of the new epidermis with little amount of granulation tissue and a higher amount of vascularized connective tissue deposition filling wound gap than in the infected untreated group. In addition, the infected wound treated with povidone-iodine showed completed re-epithelization with a slightly separated new epidermis and full amount of well-organized mature connective tissue deposition filling wound gap with obvious contraction of wound gap. The current data showed the ability of OligoF to accelerate the wound healing process reduce inflammation and eliminate infection in the injured area. The effect of OligoF on infected injured skin was comparable to povidone-iodine.

Sirtuin 1 (SIRT1) is a member of the mammalian sirtuin family^8,9^. SIRT1 regulates various biological processes and plays a critical role in regulating inflammation as well as epidermal differentiation during wound healing^10^. At the end of our experiment, the concentration and the gene expression of *SIRT1* in wounded area were measured using ELISA and real-time PCR, respectively. Treatment of non-infected wound with OligoF significantly increased the concentration and gene expression of *SIRT1* in wounded area compared to the untreated wound in the positive control group 1 (*p* < 0.001). Moreover, the concentration and gene expression of *SIRT1* increased significantly in infected wound treated with OligoF or povidone-iodine compared to untreated infected wound in the positive control group 2 (*p* < 0.001). In support of our finding, Wang et al.^33^ reported that *SIRT1* regulates oxidative stress caused by *P. aeruginosa* lipopolysaccharides in alveolar epithelial cells.

Autophagy is a self-renewal mechanism that degrades and recycles intracellular components to maintain cells ability to overcome unfavorable environments^4^. Previous studies revealed that autophagy plays a critical role in different phases of wound healing^5^. Activation of autophagy during the inflammatory phase removes infection and attenuates the inflammatory response, which helps in preventing excessive inflammation from causing further tissue damage. Moreover, induction of autophagy in the proliferative phase prevents apoptosis as well as oxidative stress and promotes cell survival^6,7^.

The more pronounced upregulation of SIRT1 and autophagy-related genes by OligoF compared to povidone-iodine suggests a mechanistic distinction between the two treatments. While povidone-iodine primarily exerts antimicrobial effects, OligoF appears to enhance host cellular repair pathways by activating autophagy, reducing oxidative stress, and promoting keratinocyte migration. This dual action may explain the superior wound healing observed with OligoF and highlights its potential as a bioactive agent targeting both pathogen control and host tissue regeneration.

Local hypoxia in the wounded area induces autophagy. Moreover, autophagy induces wound angiogenesis as well as differentiation, proliferation and migration of keratinocytes leading to wound re-epithelialisation. In the remodeling phase, autophagy of fibroblasts affects the hypertrophic scars development^5^.

Autophagy-related 5 (ATG5) and beclin 1 are essential regulatory proteins in autophagy. ATG5 has an important role in regulating mitochondrial oxidative damage as well as microglial inflammation. Moreover, BECN1 protein (beclin 1) mediates autophagy initiation and formation of autophagosomes^34^.

At the end of our experiment, the gene expression of autophagy markers (*Beclin 1* and *ATG5*) in wounded area were determined using real-time PCR. Our data showed that, treatment of non-infected wound with OligoF significantly increased the gene expression of *beclin 1* and *ATG5* (*p* < 0.0001) in the wounded area compared to untreated wound in the positive control group 1. Moreover, treatment of infected wound with OligoF or povidone-iodine significantly increased (*p* < 0.0001) the gene expression of beclin 1 and ATG5 in the wounded area compared to untreated infected wound in the positive control group 2.

In the current study, the gene expression of *SIRT1, beclin 1,* and *ATG5* in infected wound treated with povidone-iodine was significantly higher (*p* < 0.05) than their gene expression in infected wound treated with OligoF. The effect of povidone-iodine on upregulating SIRT1 and inducing autophagy in injured skin was significantly higher than OligoF. In support with our finding a recent study conducted by Chen et al.^35^ revealed that activation of SIRT1-dependent autophagy promotes differentiation and migration of the epidermal cells during wound healing process.

Histopathological analysis further supported the molecular and wound closure findings. In non-infected wounds, OligoF treatment resulted in separation of new epidermis with granulation tissue and a moderate amount of vascularized connective tissue filling the wound gap, whereas in infected wounds, OligoF promoted granulation tissue formation with increased connective tissue deposition compared to untreated controls. Povidone-iodine treatment, in contrast, induced complete re-epithelialization with well-organized mature connective tissue and clear wound contraction. These observations indicate that while both treatments accelerate healing, OligoF primarily enhances tissue regeneration through granulation and connective tissue formation, whereas povidone-iodine’s effects are more pronounced on epidermal closure. Integrating these findings with the upregulation of SIRT1 and autophagy-related genes suggests that OligoF may facilitate wound repair through activation of host cellular pathways, in addition to its antibacterial properties.

It should be noted that direct assessment of OligoF’s stability, pharmacokinetics, and bioavailability was not performed in the current preclinical study. Future studies are warranted to evaluate these parameters to establish clinical relevance and optimize dosing strategies.

In conclusion, OligoF accelerated the wound healing process and its effect was comparable to povidone-iodine in enhancing the healing of infected wound. The beneficial effect of OligoF on wound healing could be due to upregulation of *SIRT1* and induction of autophagy. Further clinical studies are recommended for the effect of OligoF on the healing of infected wounds.

## Supplementary Information

Below is the link to the electronic supplementary material.

Supplementary Material 1

## Data Availability

Data is provided within the manuscript or supplementary information files.
