# Supplementary material for: SIRT1 mediated autophagy enhancement by Lactobacillus fermentum derived oligosaccharides accelerates wound healing in biofilm associated infection
Source: Sci Rep. 2025 Dec 26;15:44667. doi: 10.1038/s41598-025-30280-2 (PMC12748636; doi:10.1038/s41598-025-30280-2)
Supplement: Supplementary file 1 — Supplementary Material 1 [file 41598_2025_30280_MOESM1_ESM.pdf]

Supporting data for the manuscript entitled:

SIRT1 Mediated Autophagy Enhancement by *Lactobacillus fermentum* Derived Oligosaccharides Accelerates Wound Healing in Biofilm Associated Infection

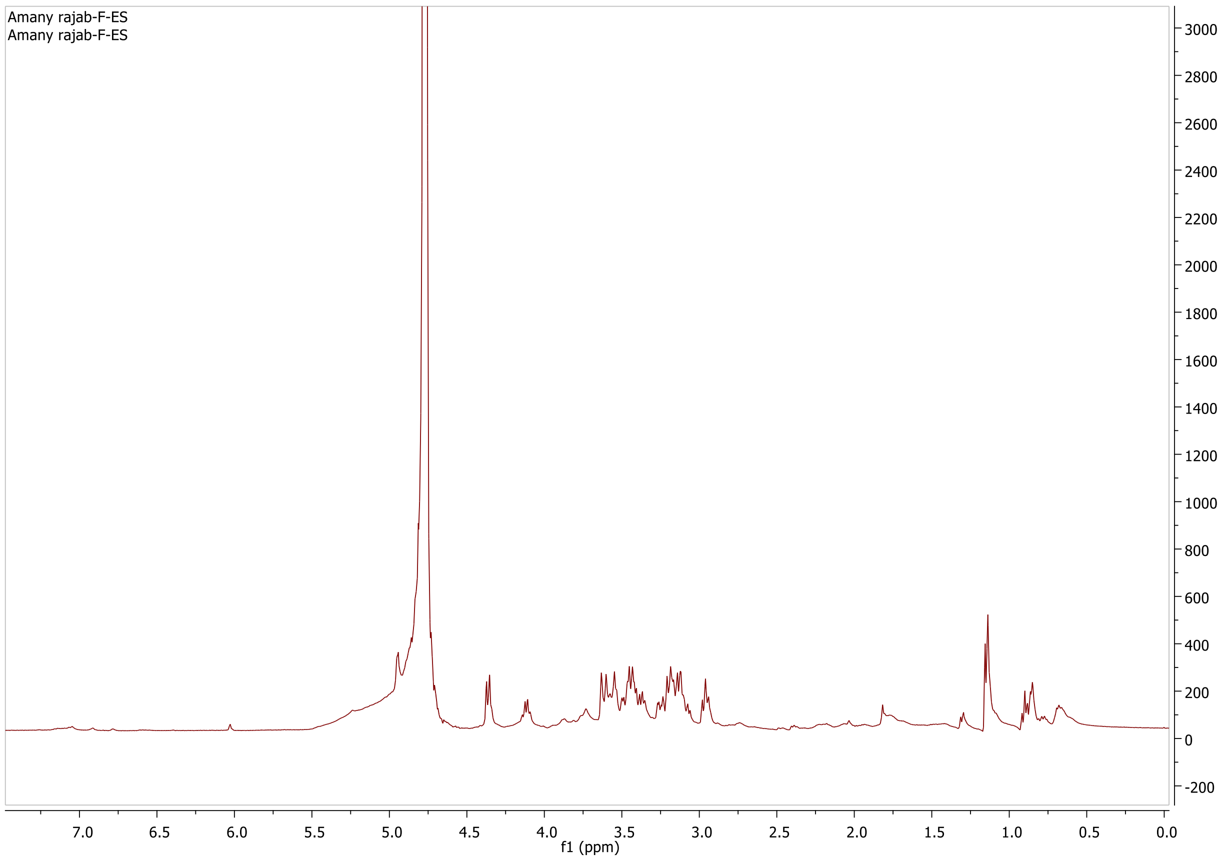

Figure S1: ^{1}H NMR spectrum of OligoF (400 MHz, D_2O)

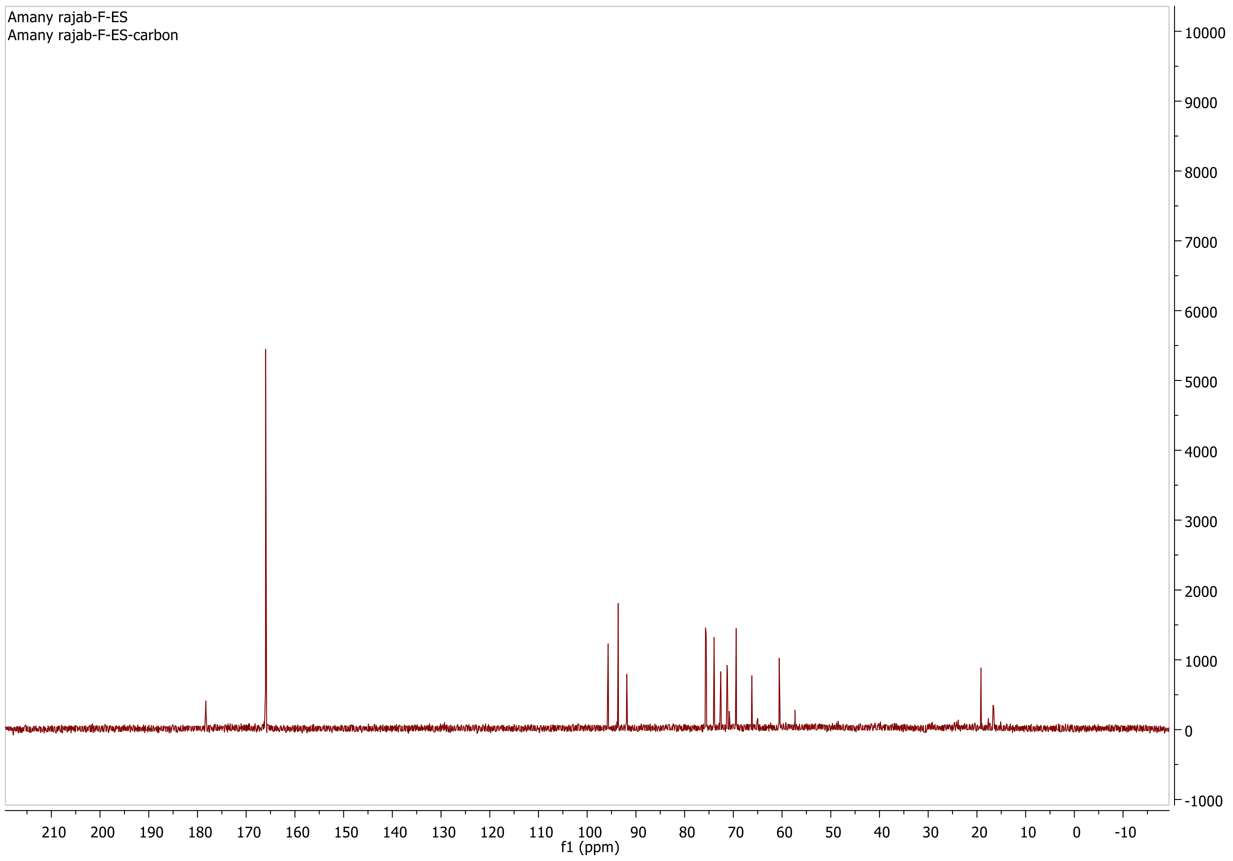

Figure S2: ^{13}C NMR spectrum of OligoF (125 MHz, D_2O)

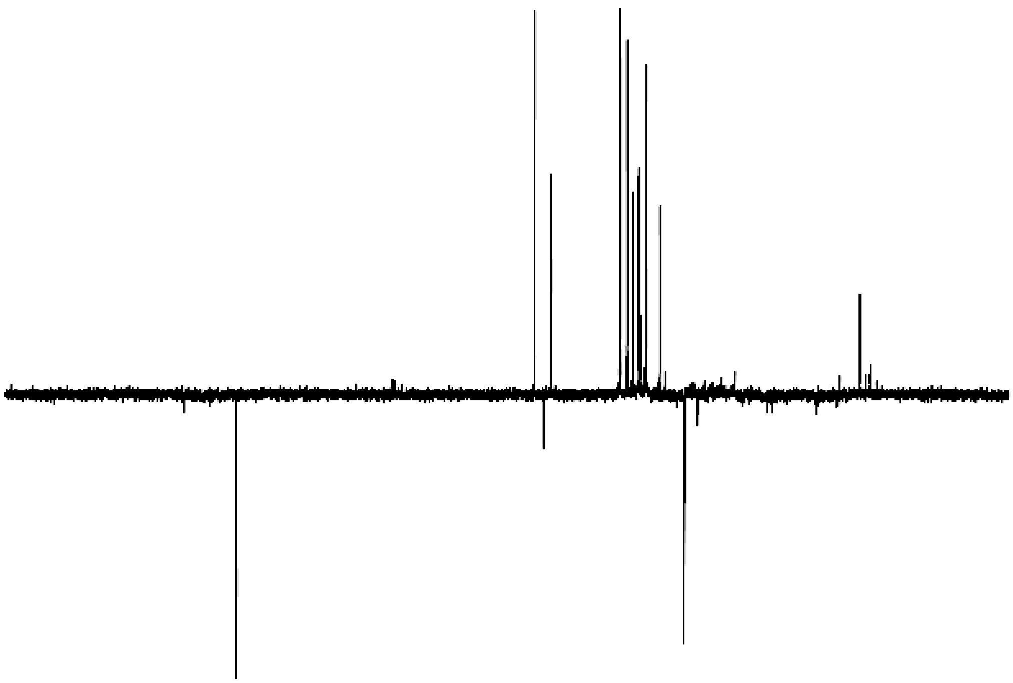

Figure S3: DEPTQ NMR spectrum of OligoF (DMSO-d_6)

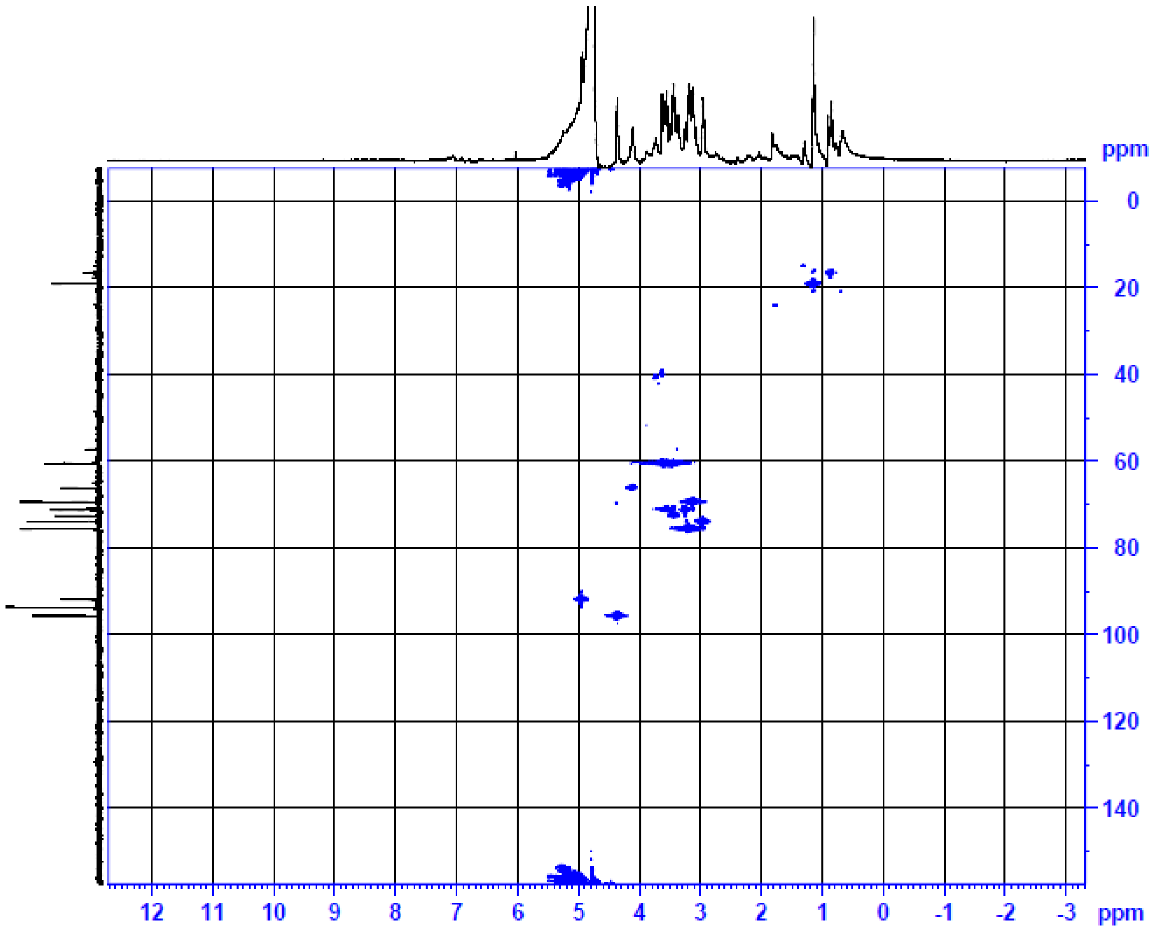

Figure S4: HSQC NMR spectrum of OligoF (D_2O)

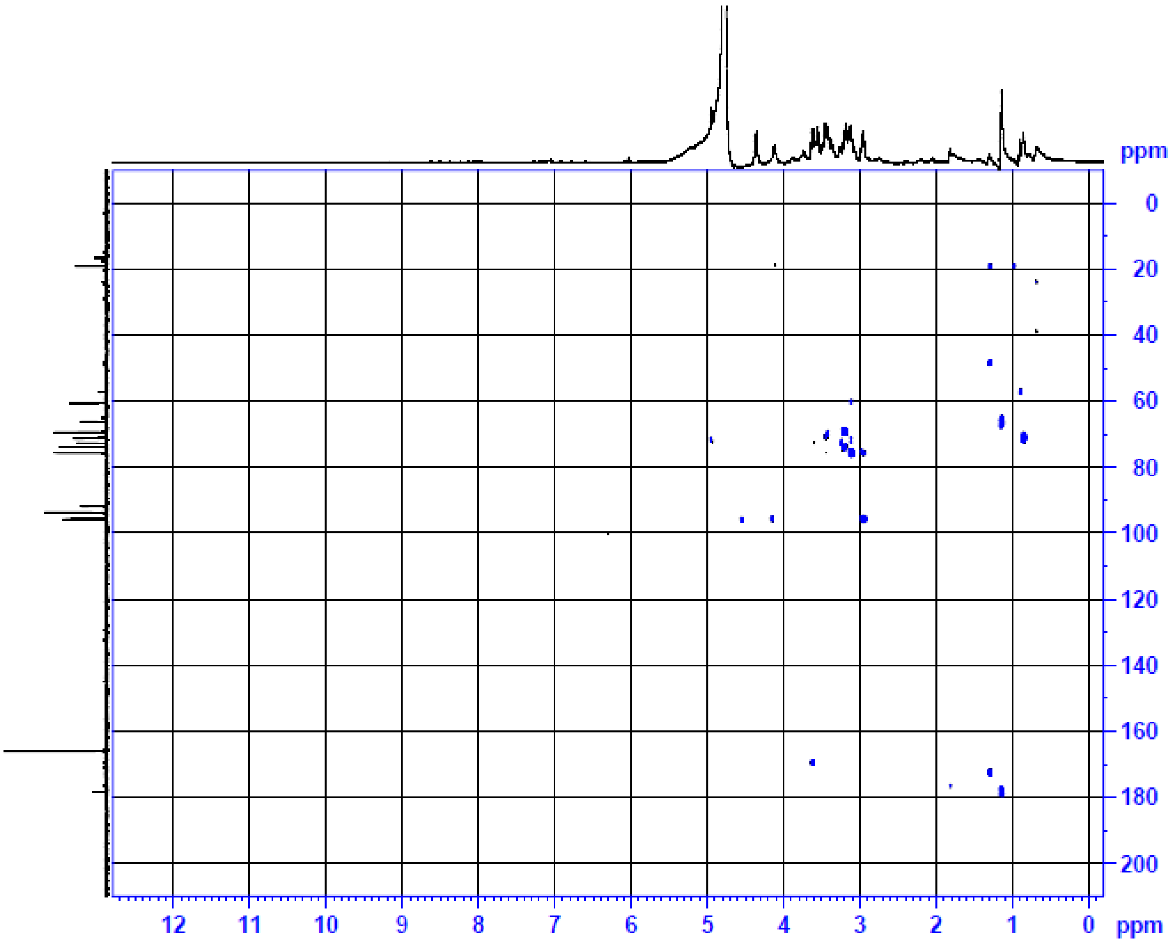

Figure S5: HMBC NMR spectrum of OligoF (D_2O)

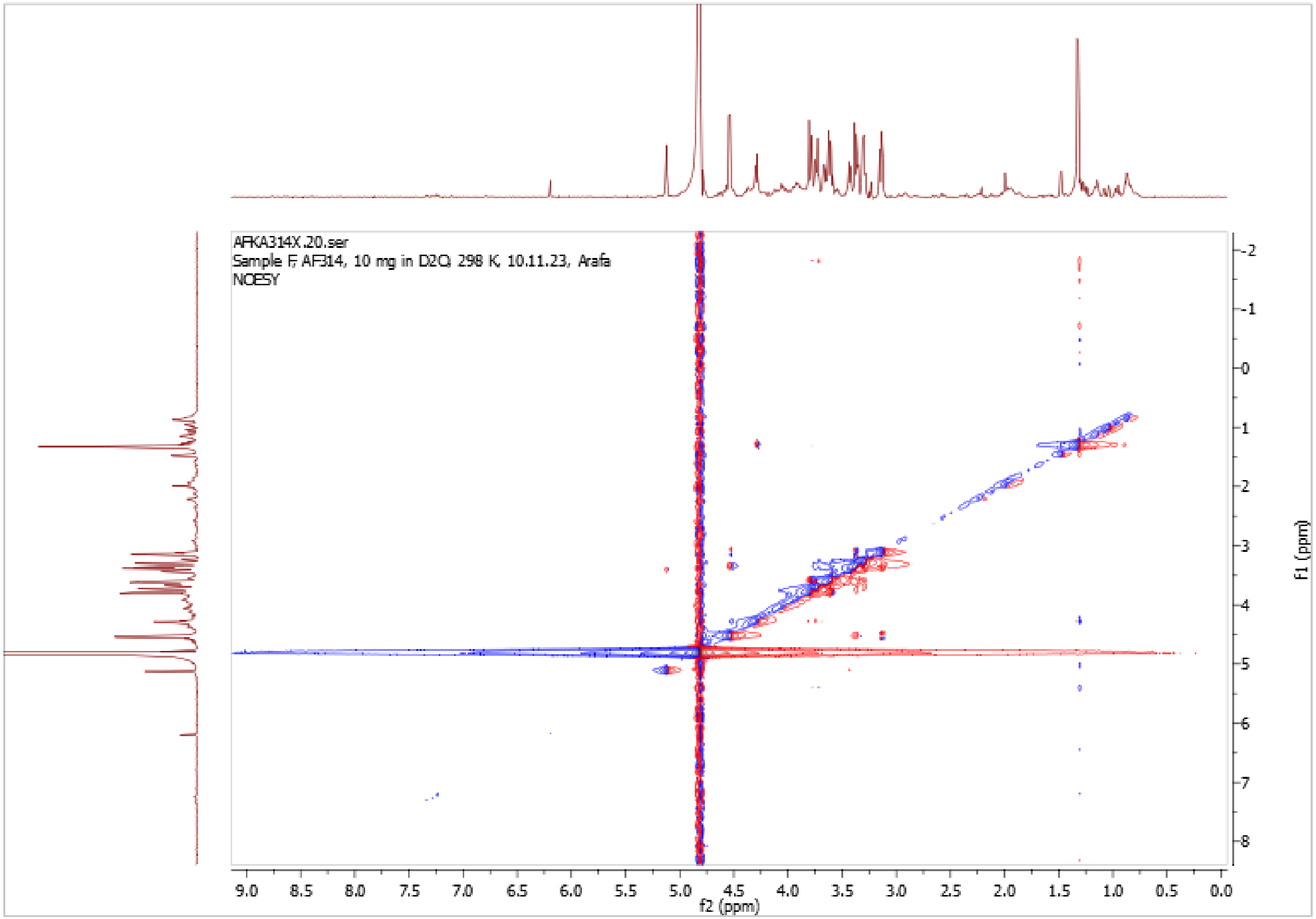

Figure S6: NOESY NMR spectrum of OligoF.
